# Super-enhancer-driven lncRNA LIMD1-AS1 activated by CDK7 promotes glioma progression

**DOI:** 10.1038/s41419-023-05892-z

**Published:** 2023-06-29

**Authors:** Zhigang Chen, Dasheng Tian, Xueran Chen, Meng Cheng, Han Xie, JiaJia Zhao, Jun Liu, Zhiyou Fang, Bing Zhao, Erbao Bian

**Affiliations:** 1 Department of Neurosurgery, the Second Affiliated Hospital of Anhui Medical University, 678 Fu Rong Road, Hefei, Anhui 230601 China; 2 Cerebral Vascular Disease Research Center, Anhui Medical University, 678 Fu Rong Road, Hefei, Anhui 230601 China; 3 Department of Orthopaedics, the Second Affiliated Hospital of Anhui Medical University, 678 Fu Rong Road, Hefei, Anhui 230601 China; 4 Department of Laboratory Medicine, Hefei Cancer Hospital, Chinese Academy of Sciences, No. 350, Shushan Hu Road, Hefei, Anhui 230601 China; 5 Anhui Province Key Laboratory of Medical Physics and Technology; Institute of Health and Medical Technology, Hefei Institutes of Physical Science, Chinese Academy of Sciences, No. 350, Shushan Hu Road, Hefei, Anhui 230601 China

**Keywords:** Cancer, Molecular biology

## Abstract

Long non-coding RNAs (lncRNAs) are tissue-specific expression patterns and dysregulated in cancer. How they are regulated still needs to be determined. We aimed to investigate the functions of glioma-specific lncRNA LIMD1-AS1 activated by super-enhancer (SE) and identify the potential mechanisms. In this paper, we identified a SE-driven lncRNA, LIMD1-AS1, which is expressed at significantly higher levels in glioma than in normal brain tissue. High LIMD1-AS1 levels were significantly associated with a shorter survival time of glioma patients. LIMD1-AS1 overexpression significantly enhanced glioma cells proliferation, colony formation, migration, and invasion, whereas LIMD1-AS1 knockdown inhibited their proliferation, colony formation, migration, and invasion, and the xenograft tumor growth of glioma cells in vivo. Mechanically, inhibition of CDK7 significantly attenuates MED1 recruitment to the super-enhancer of LIMD1-AS1 and then decreases the expression of LIMD1-AS1. Most importantly, LIMD1-AS1 could directly bind to HSPA5, leading to the activation of interferon signaling. Our findings support the idea that CDK7 mediated-epigenetically activation of LIMD1-AS1 plays a crucial role in glioma progression and provides a promising therapeutic approach for patients with glioma.

## Introduction

Long non-coding RNAs (lncRNAs) are identified as non-coding transcripts with a length of more than 200 nucleotides and a lack of protein-coding ability [1]. LncRNAs exert biological functions through various molecular mechanisms based on subcellular location. In the nucleus, lncRNAs can act in cis to regulate neighboring gene expression or in trans to control long-distance gene expression [2, 3]. In contrast, cytoplasmic lncRNAs often regulate gene expression at the post-transcriptional level through regulating mRNA or protein stability, its subcellular localization, or serving as competing with endogenous-RNA (ceRNA) [4, 5]. Recently, other research groups and we have revealed that several lncRNAs, including HOTAIR, MEG3, ATB, and H19, play a critical role in the initiation and progression of glioma [6–9]. Although RNA sequencing analyses have identified many novel lncRNAs in glioma, their regulatory mechanisms and biological functions still need to be thoroughly investigated. Therefore, the identification of novel glioma-associated lncRNAs is essential for the discovery of effective therapeutic targets.

Enhancers are non-coding regulatory elements that regulate gene transcription through looping-mediated interactions with promoters and are activated in specific cellular contexts by recruiting transcription factors [10]. Super enhancers (SEs) are referred to as clusters of enhancers, characterized by a high degree of enrichment of master transcription factors and mediator coactivators, that drive the tissue-specific gene expression [11]. SEs are found to drive the expression of genes that play prominent roles in regulating cell identity during development and tumorigenesis [12, 13]. In addition, SEs can also drive the expression of lncRNAs with tumor-promoting functions. For example, SEs-associated lncRNAs such as LINC01503 in squamous cell carcinoma (SCC), lncRNA HCCL5 in Hepatocellular carcinoma (HCC), lncRNA UCA1 in Epithelial Ovarian Cancer, have been revealed in cancer [14, 15]. Thus, it would be essential to explore the potential involvement of SE-associated oncogenic lncRNAs in the pathogenesis of glioma. Increasing evidence has shown that cancer cells are particularly sensitive to the inhibition of the SE-complex, which consequently causes selective inhibition of SE-driven transcription [16]. Therapeutically, inhibitors targeting the SE-complex have also shown promising anti-cancer activity in several pre-clinical models [17, 18].

In this study, we identified the SE-driven-LIMD1-AS1 in glioma cells by H3K27ac and MED1 ChIP-seq and SE-driven-LIMD1-AS1 high expression in glioma tissues and correlated with shorter survival times of patients. Functionally, LIMD1-AS1 exhibits promotion effects on the malignant phenotype of glioma. Mechanically, CDK7 interacts with MED1 to regulate the LIMD1-AS1 super-enhancer. In addition, we found that glioma cells are extremely susceptible to the inhibitor of CDK7, as this inhibition represses SE-driven-LIMD1-AS1. Finally, LIMD1-AS1 binds to HSPA5, followed by activation of interferon signaling.

## Results

### Identification of novel SE-associated oncogenic lncRNAs in glioma

**Fig. 1:**
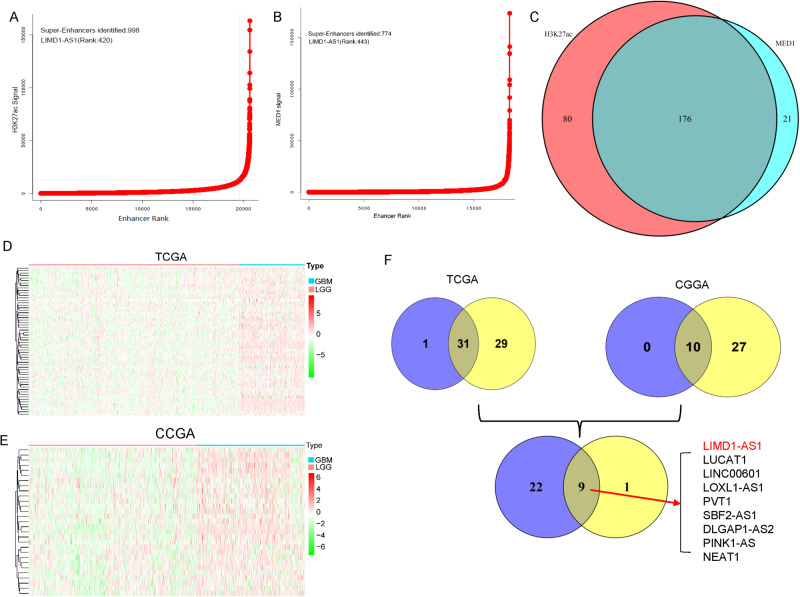
The SE-driven-LIMD1-AS1 is uniquely upregulated in glioma. **A**, **B** Hockey stick plots showing rank order of H3K27ac and MED1 signals for all enhancers in GBM cells. **C** The overlapping SE-related lncRNAs are co-occupied by H3K27ac and MED1. **D**, **E** The expression of SE-lncRNAs in GBM vs LGG from TCGA and CGGA database. **F** Venn diagram showing the intersection between SE-lncRNAs for which high expression is associated with poor patient prognosis as calculated by the Cox proportional hazard test and the Log-rank test.

**Fig. 2:**
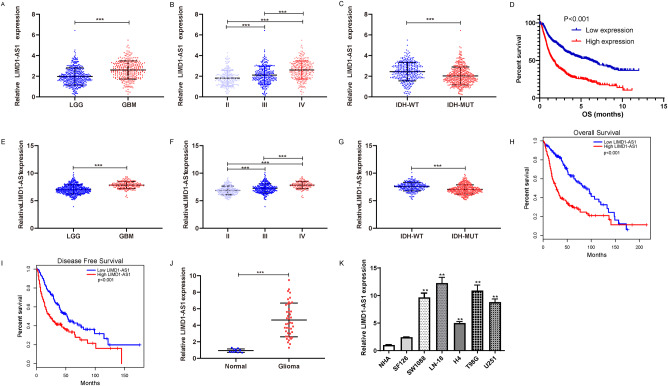
Correlation between LIMD1-AS1 expression and the clinic-pathologic features of gliomas. **A** The LIMD1-AS1 expression is shown in GBM vs LGG in CGGA. ***P < 0.001. **B** The expression of LIMD1-AS1 in different glioma grades. ***P < 0.001. **C** The expression of LIMD1-AS1 is significantly associated with IDH mutation in CGGA glioma. ***P < 0.001. **D** Prognostic significance of LIMD1-AS1 in CGGA gliomas. **E** The LIMD1-AS1 expression is shown in GBM vs LGG in TCGA. ***P < 0.001. **F** The expression of LIMD1-AS1 in different glioma grades. ***P < 0.001. **G** The expression of LIMD1-AS1 is significantly associated with IDH mutation in TCGA glioma. *P < 0.05. **H**, **I** Prognostic significance of LIMD1-AS1 in gliomas. **J** The LIMD1-AS1 expression in glioma and normal brain tissues from our cohort. ***P < 0.001. **K** Relative LIMD1-AS1 in a panel of glioma cell lines and NHA. **P < 0.01.

A previous study has demonstrated that SE-associated protein-coding genes were characterized in integrative analysis of both sensitivities to transcriptional inhibition and their expression levels in glioma cells [19]. To investigate the implications of SE-associated lncRNAs in glioma, the publicly available H3K27Ac and MED1 ChIP-seq datasets of glioblastoma (GBM) cells were obtained and analyzed to generate a catalog of SEs (Fig. 1A, B). We identified 256 and 197 SE-associated lncRNAs upon occupancy of either H3K27Ac or MED1, respectively (Fig. 1C). Importantly, 176 SE-associated lncRNAs were overlapped (Fig. 1C). To prioritize these SE-associated lncRNAs for further study, we selected lncRNAs with (1) elevated expression in GBM tissue versus low-grade glioma (LGG) tissue (Fig. 1D, E) and (2) prognostic significance from the integrated analysis CGGA and TCGA databases, where high lncRNA expression was associated with poor glioma patient survival (Fig. 1F). A total of nine differentially expressed SE-lncRNAs were selected for further analyses (Fig. 1F). To validate data, we analyzed nine candidates’ SE-lncRNAs expression in the GEPIA database. As shown in Supplementary Fig. 1A–F, LUCAT1, LINC00601, LOXL1-AS1, SBF2-AS1, PINK1-AS, and NEAT1 were observed with no significant differences in glioma compared to normal tissues. PVT1 and DLGAP1-AS2 were highly expressed in GBM but not LGG compared to normal brain tissue (Supplementary Fig. 1G–H). Notably, only LIMD1-AS1 was finally found to be significantly overexpressed in GBM and LGG, but in other types of cancer, suggesting that LIMD1-AS1 may be mainly involved in the progression of glioma (Supplementary Fig. 1I).

### LIMD1-AS1 is an oncogenic lncRNA in glioma

To study the roles of LIMD1-AS1 in gliomas, two published datasets: CGGA and TCGA, were analyzed. In CGGA datasets, LIMD1-AS1 was highly expressed in GBM samples compared to that in LGG and associated with the degree of malignancy, IDH mut (Fig. 2A–C). The elevated level of LIMD1-AS1 expression indicated poor survival for glioma patients (Fig. 2D). Consistent with these results, the expression of LIMD1-AS1 in gliomas was related to the degree of malignancy, IDH mut, and associated with poor survival for glioma patients in the TCGA database (Fig. 2E–I). Our cohort confirmed that LIMD1-AS1 had a higher expression in gliomas than in normal brain tissue (Fig. 2J). RT-qPCR analysis showed that LIMD1-AS1 expression was higher in all 4 GBM compared with LGG and normal astrocyte cell lines (Fig. 2K). Therefore, LIMD1-AS1 might exert an essential function in glioma progression.

We next explored the underlying mechanism by which LIMD1-AS1 is upregulated in glioma. Abnormal activation of proto-oncogenes caused by genomic gain, amplification, and hypomethylation is frequent in gliomas [20, 21]. Firstly, we analyzed the DNA methylation data of the LIMD1-AS1 gene in glioma from the TCGA database. There was no significant association between LIMD1-AS1 methylation and its expression (Supplementary Fig. 2A, B). We then focused on the copy number variation (CNV) data of glioma in TCGA as previously described [22]. Although the copy number of LIMD1-AS1 was frequently upregulated in GBM, the copy number of LIMD1-AS1 slightly correlated with LIMD1-AS1 expression in glioma samples (Supplementary Fig. 2C, D).

**Fig. 3:**
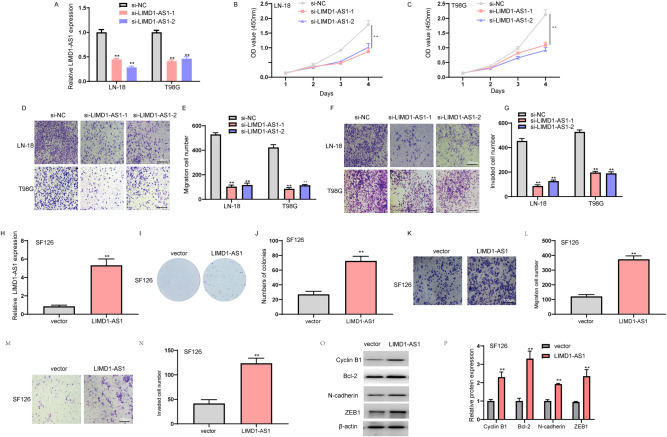
The effect of LIMD1-AS1 on the malignant phenotype of glioma cells. **A** Expression of LIMD1-AS1 in LN-18 and T98G cells transfected si-LIMD1-AS1 as measured by RT-qPCR. **P < 0.01. **B**, **C** CCK8 assays were performed to examine the proliferation in LN-18 and T98G cells transfected with two different siRNAs. **P < 0.01. **D**, **E** Transwell assays were performed to observe the number of migrated cells in LN-18 and T98G cells transfected with two different siRNAs. **P < 0.01. **F**, **G** Transwell assays were performed to observe the number of invaded cells in LN-18 and T98G cells transfected with two different siRNAs. **P < 0.01. **H** LIMD1-AS1 expression in SF126 cells transfected LIMD1-AS1 plasmid as measured by qPCR. **P < 0.01. **I**, **J** LIMD1-AS1 overexpression cells increased clone formation ability. **P < 0.01. **K**–**N** Transwell assays were performed to examine the number of migrated or invaded cells in SF126 cells transfected with LIMD1-AS1 plasmid. **P < 0.01. **O**–**P** Western blotting results for the protein level of proliferation and EMT-associated markers in SF126 glioma cells transfected with vector/LIMD1-AS1. **P < 0.01.

To explore the cellular function of LIMD1-AS1, we performed the loss or gain-of-function assay to study its biological role in glioma cells. Specifically, siRNAs were applied to LN-18 and T98G glioma cell lines exhibiting high LIMD1-AS1 expression levels, while an overexpression plasmid was used in low-expressing LIMD1-AS1 SF126 glioma cells (Figs. 2K, 3A). LIMD1-AS1 knockdown by siRNA significantly inhibited cell viability in both LN-18 and T98G glioma cells, while LIMD1-AS1 overexpression increased clonogenic ability of SF126 glioma cells (Fig. 3B, C, H–J). Importantly, LIMD1-AS1 knockdown markedly reduced the migration and invasion in T98G and LN-18 (Fig. 3D–G), while ectopic expression of LIMD1-AS1 promoted migration and invasion in SF126 glioma cells (Fig. 3K–N). Accordingly, western blotting showed that overexpression of LIMD1-AS1 resulted in higher Cyclin B1, Bcl-2, N-cadherin, and ZEB1 expression at protein levels in SF126 glioma cells (Fig. 3O, P). These data identified LIMD1-AS1 as a functionally oncogenic lncRNA in glioma.

**Fig. 4:**
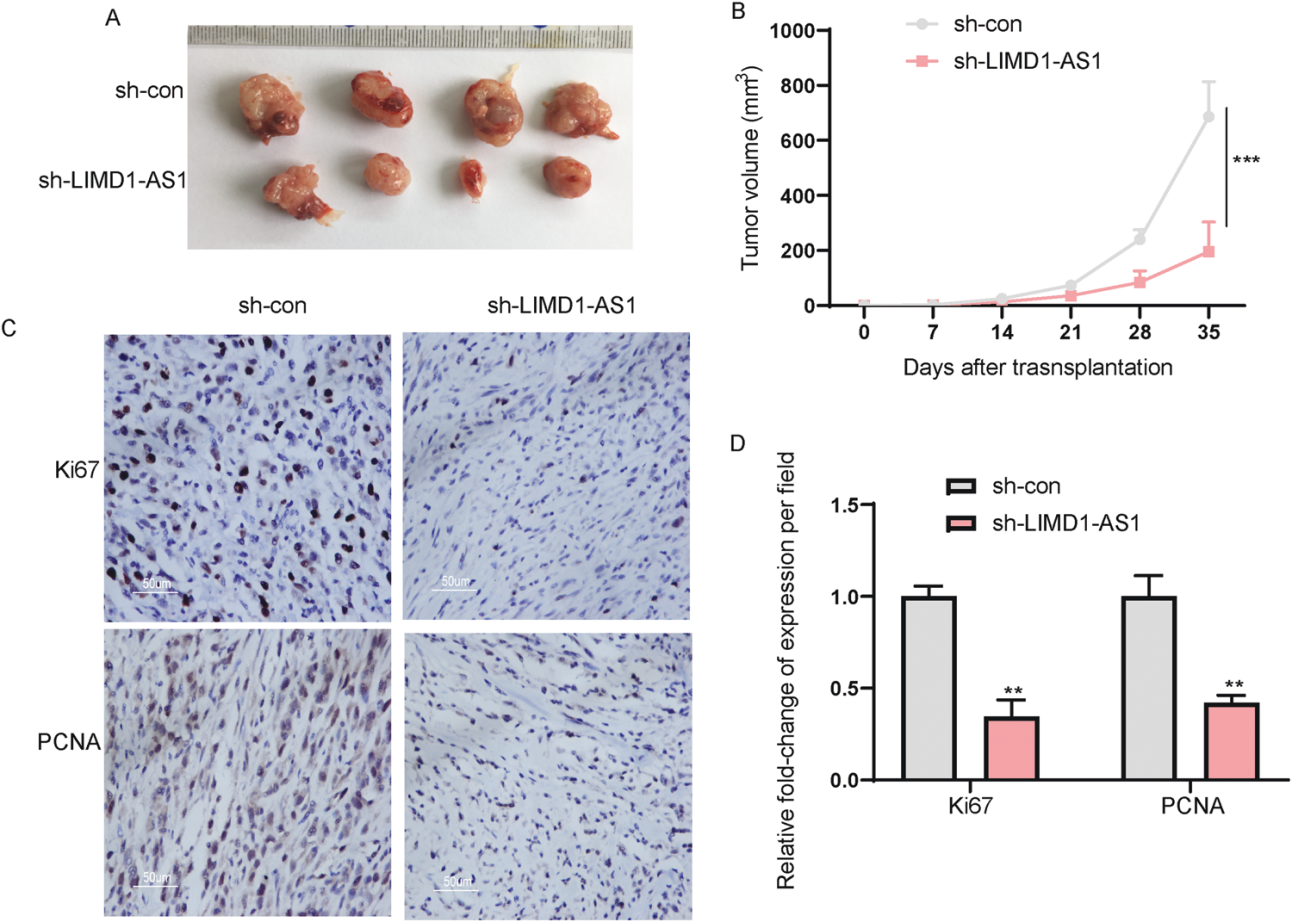
Targeting LIMD1-AS1 attenuates in vivo tumor growth. **A**, **B** Knockdown of LIMD1-AS1 attenuated subcutaneous tumor growth. ***P < 0.001, as compared with sh-con cells. **C**, **D** Representative ki-67 and PCNA IHC staining in mouse xenografts after sh-LIMD1-AS1 treatment. **P < 0.01.

To examine the function of LIMD1-AS1 in vivo, glioma cells were implanted into nude mice subcutaneously. Knockdown of LIMD1-AS1 dramatically reduced tumor growth in xenograft mouse tumor models (Fig. 4A, B). In support of the pro-tumor role of LIMD1-AS1, the Ki67 and PCNA staining showed that LIMD1-AS1 knockdown reduced tumor cell proliferation in vivo (Fig. 4C, D). Our data suggest that LIMD1-AS1 contributes to glioma progression.

### LIMD1-AS1 is a SE-driven lncRNA in glioma

**Fig. 5:**
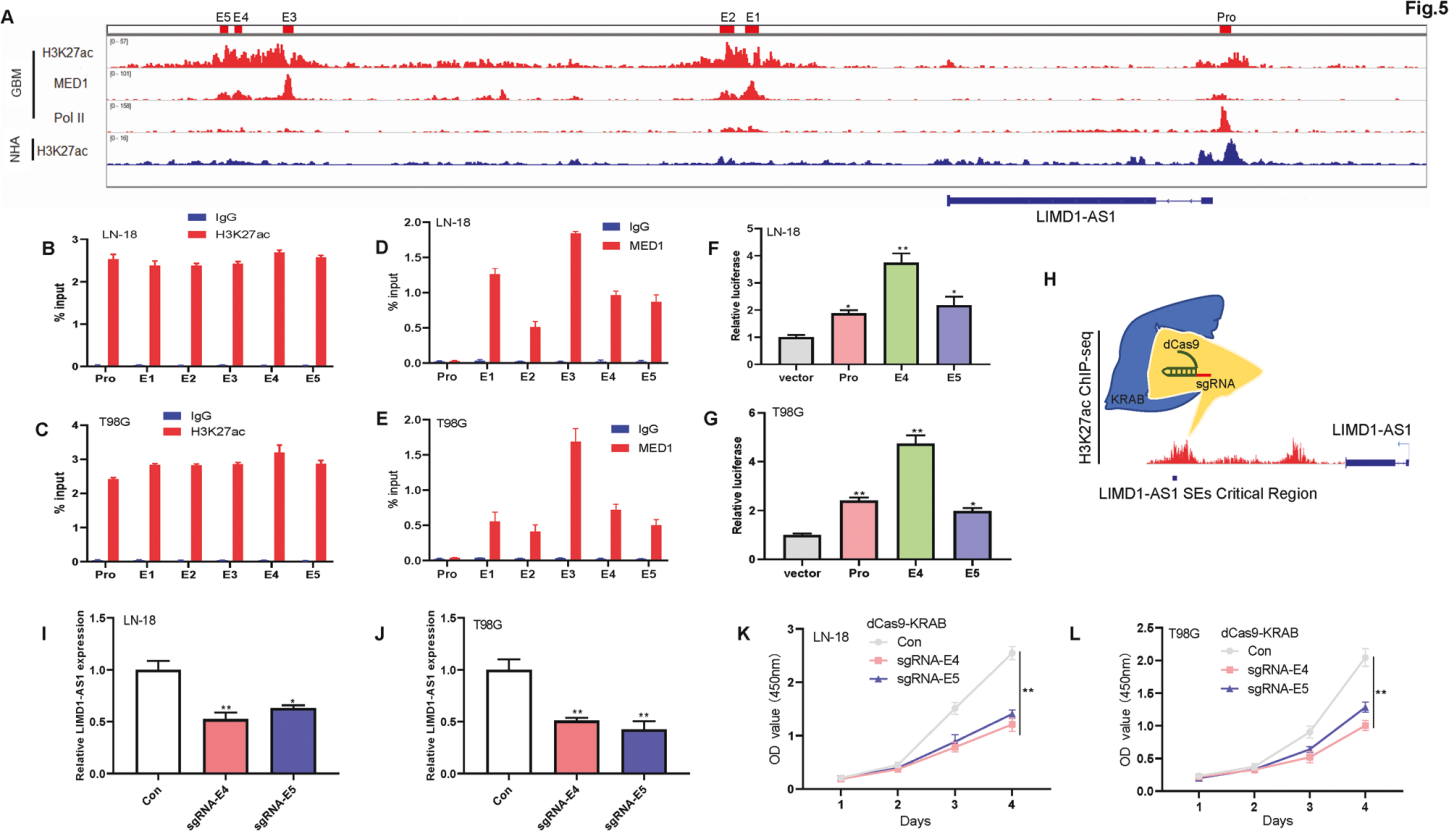
The identity of SE-driven LIMD1-AS1 in glioma. **A** Genome browser image at Chip-seq profiles for H3K27ac, MED1, Pol II at LIMD1-AS1 locus in GBM cells and NHA cell lines. **B**, **C** ChIP-qPCR analysis of the H3K27ac enrichment in the LIMD1-AS1 promoter and constituent enhancer regions. IgG antibody was used as a negative control. **D**, **E** ChIP-qPCR analysis of the MED1 enrichment in the LIMD1-AS1 promoter and constituent enhancer regions. IgG antibody was used as a negative control. **F**, **G** Luciferase reporter assays measured Promoter and Enhancer (E4, E5) activity of LIMD1-AS1 in LN-18 and T98G glioma cells. *P < 0.05, **P < 0.01. **H** sgRNAs designed to target the LIMD1-AS1 super-enhancer critical region using the dCas9-KRAB. **I**, **J** Blockade of LIMD1-AS1 super-enhancer critical region by two individual sgRNAs significantly reduced the expression of LIMD1-AS1. *P < 0.05, **P < 0.01. **K**, **L** Blockade of LIMD1-AS1 super-enhancer critical region by two individual sgRNAs reduced the proliferation in LN-18 and T98G glioma cells. **P < 0.01.

To characterize the transcriptional regulation of LIMD1-AS1, H3K27ac, MED1, and Pol II profiles in GBM cell lines were analyzed by ChIP-seq. An SE upstream of LIMD1-AS1 was found in GBM cell lines but was absent in normal human cell lines (Fig. 5A). In addition, co-localized with H3K27ac and MED1 (known to identify enhancer elements) in the identified super-enhancer of LIMD1-AS1 (Fig. 5A). To further verify H3K27ac and MED1 binds the SE and promoter of LIMD1-AS1, ChIP analysis was performed to show that H3K27ac binds SE and promoter of LIMD1-AS1 in LN-18 and T98G glioma cells (Fig. 5B, C). As expected, MED1 binds only at the LIMD1-AS1 super-enhancer (Fig. 5D, E). We next cloned promoter and individual super-enhancer constituents (E4 and E5) of the LIMD1-AS1 into promoter-reporter and enhancer-reporter vectors and then measured their activities by luciferase reporter assay. E4, E5, and promoter were active in LN-18 and T98G glioma cell lines (Fig. 5F, G). As shown in Fig. 5H–J, the recruitment of the dCas9-KRAB to interfere with LIMD1-AS1 SEs regions resulted in significant downregulation of LIMD1-AS1 expression in LN-18 and T98G glioma cells. Silencing of the super-enhancer component decreased the proliferation in LN-18 and T98G glioma cells (Fig. 5K, L). Collectively, these results suggest that increased LIMD1-AS1 is driven by super-enhancers to promote glioma cell growth.

### CDK7 interacts with MED1 to regulate the super-enhancer of LIMD1-AS1

**Fig. 6:**
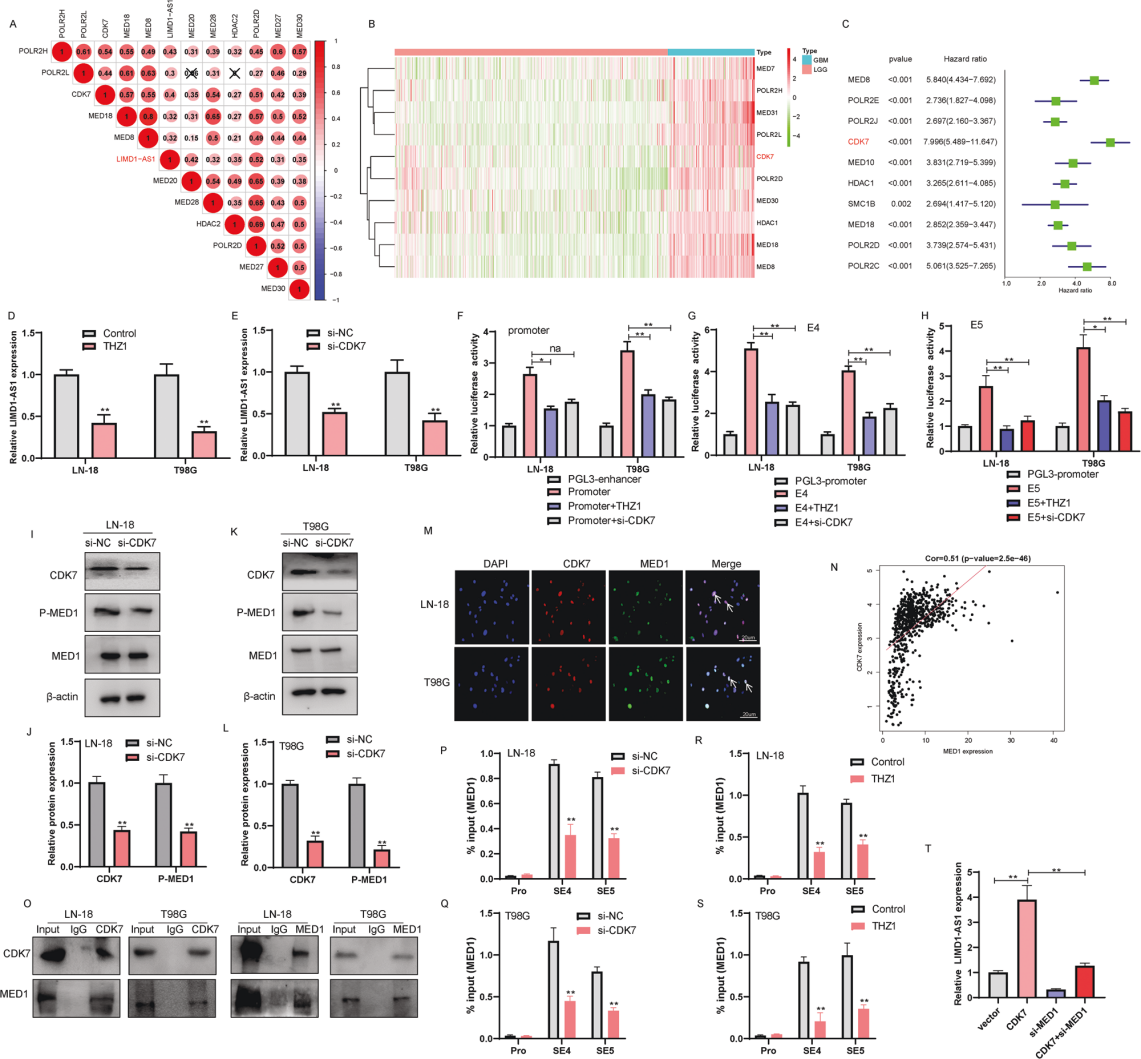
The interaction of CDK7 with MED1 regulates LIMD1-AS1 transcription in glioma. **A** The correlation between LIMD1-AS1 and SE complexes in glioma tissues from the TCGA database. **B** Heatmap of differentially expressed SE complexes between GBM and LGG. **C** Univariate analysis presents the hazard ratios and *P*-value of SE complexes-related genes by the forest plot. **D**, **E** Expression of LIMD1-AS1 in LN-18 and T98G cells transfected si-CDK7 or treated by THZ1 as measured by RT-qPCR. **P < 0.01. **F**–**H** LIMD1-AS1 promoter and enhancer activities are measured by luciferase reporter assays. Luciferase activity is reduced by THZ1 treatment or CDK7 knockdown in LN-18 and T98G glioma cells. *P < 0.05, **P < 0.01. **I**–**L** LN-18 and T98G glioma cells were either transfected with si-NC/si-CDK7 followed by western blot analysis with the mentioned antibodies. **M** The CDK7 co-localized with MED1 in LN-18 and T98G glioma cells were analyzed by IF experiments. **N** The expression of CDK7 is significantly associated with MED1 expression in the CCGA database. **O** Reciprocal CO-IP analysis from LN-18 and T98G glioma cells with MED1 and CDK7 specific antibodies, demonstrating the interaction between MED1 and CDK7. **P**–**S** ChIP-qPCR showed that inhibition of CDK7 significantly reduced the occupancy of MED1 on the SE region of LIMD1-AS1. **T** RT-qPCR were performed to observe the LIMD1-AS1 expression in SF126 glioma cells transfected with CDK7 plasmid or/and si-MED1. **P < 0.01.

SE-complex involved in the effect of SE functions as independent or interdependent components of these large transcription-regulating complexes to drive high-level expression of their associated gene [23–25]. Thus, we explored whether the components of the SE-complex are involved in the regulation of LIMD1-AS1 in glioma progression. To address this question, we selected SE-complex with (1) significantly correlated with the expression of LIMD1-AS1, (2) Top ten expressions in GBM tissue versus LGG tissue from integrated analyses of and (3) prognostic significance in TCGA and CGGA database, where high lncRNA expression was associated with poor glioma patient survival (Fig. 6A–C, Supplementary Fig. 3A–C). Thus, the top-ranked gene CDK7 was selected for further validation and analysis. Cyclin-Dependent Kinase 7 (CDK7) is a CDK-activating kinase (CAK), and as a component of the general transcription factor TFIIH, it mediates RNA polymerase-II-based transcription and contributes to tumor progression [26, 27]. THZ1, a CDK7 inhibitor, treatment markedly reduced LIMD1-AS1 expression in LN-18 and T98G glioma cells (Fig. 6D). Similarly, CDK7 knockdown also markedly decreased LIMD1-AS1 expression in LN-18 and T98G glioma cells (Fig. 6E, Supplementary Fig. 3D). Mechanistically, we observed that the reporter activity was prominently increased upon transfection of either the LIMD1-AS1 promoter or enhancer (E4, E5), whereas THZ1 treatment or CDK7 knockdown potently inhibited this reporter activity (Fig. 6F–H). Taken together, our findings support the hypothesis that the perturbation of SE-complex components may collectively suppress the transcriptional activation activity of LIMD1-AS1 in glioma.

A recent study showed CDK7 inhibition selectively targets MED1-mediated oncogenic transcriptional amplification [28]. The mediator complex subunit 1 (MED1) is a component of the mediator complex and functions as a transcriptional coactivator specifically enriched in a novel class of transcription regulatory DNA regions called super-enhancers [29]. Because MED1 enriched in super-enhancers of LIMD1-AS1 (Fig. 5C), we sought to determine whether CDK7 recruits MED1 to the super-enhancer of LIMD1-AS1. Firstly, the inhibition of CDK7 decreased the mRNA expression of MED1 in LN-18 and T98G glioma cells (Supplementary Fig. 3E). In addition, CDK7 knockdown reduced p-MED1 levels in LN-18 and T98G glioma cells, but had no significant effect on MED1 protein expression (Fig. 6I–L). We examined the subcellular location of CDK7 and MED1 using immunofluorescence staining and found that CDK7 mainly colocalized with MED1 in the nucleus of LN-18 and T98G glioma cells (Fig. 6M). Most importantly, CDK7 significantly correlated with MED1 expression in glioma tissues from the TCGA and CGGA database (Fig. 6N, Supplementary Fig. 3F). Furthermore, endogenous CDK7 coimmunoprecipitated with endogenous MED1 in LN-18 and T98G glioma cells (Fig. 6O). The interaction between MED1 and CDK7 was further demonstrated by reverse endogenous coimmunoprecipitation of MED1 with CDK7 (Fig. 6O), verifying the interaction between MED1 and CDK7 in vitro. These results prompted us to explore whether CDK7 is associated with super-enhancer of LIMD1-AS1 mediated by MED1, we conducted a ChIP analysis. We found that the inhibition of CDK7 decreased the binding of MED1 across the LIMD1-AS1 super-enhancer, but not the promoter, in LN-18 and T98G glioma cells (Fig. 6P–S). To further verify whether MED1 is involved in epigenetic activation of LIMD1-AS1 induced CDK7, we observed LIMD1-AS1 expression after co-transfection of si-MED1 and CDK7 to SF126 glioma cells. As shown in Fig. 6T, MED1 knockdown partially reduced LIMD1-AS1 expression induced by CDK7. Together, these data indicated that MED1 might be involved in the function of CDK7 as an epigenetic activator of LIMD1-AS1 in glioma cells.

### A covalent CDK7 Inhibitor alleviated LIMD1-AS1-promoted the growth of glioma cells

**Fig. 7:**
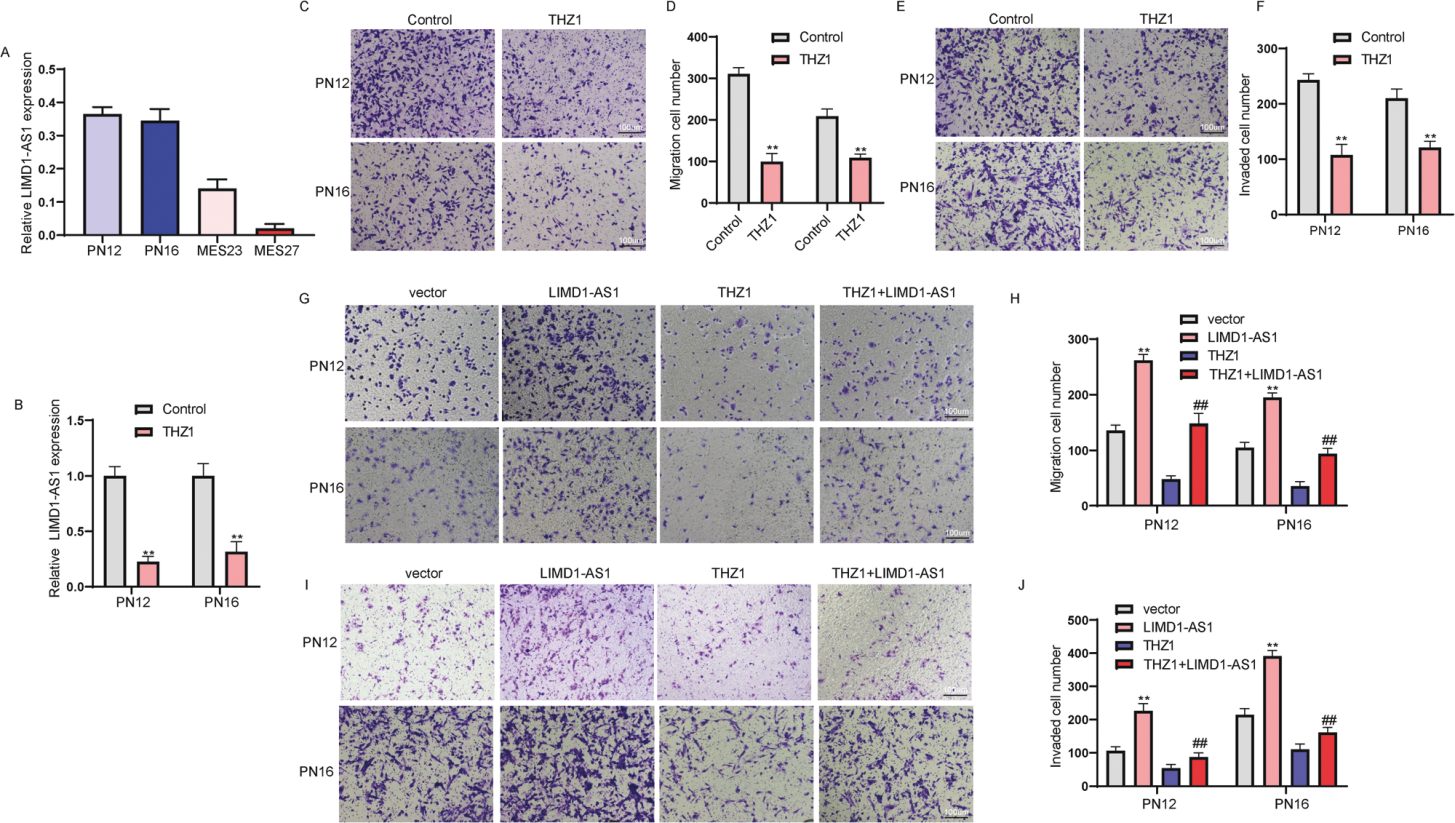
LIMD1-AS1 reversed THZ1-mediated the growth inhibition of glioma cells. **A** Expression of LIMD1-AS1 in patient-derived glioblastoma cells. **B** Expression of LIMD1-AS1 in PN12 and PN16 treated with Control or THZ1. **P < 0.01. **C**, **D** The migration of PN12 and PN16 cells treated with Control or THZ1. **P < 0.01. **E**, **F** The invasion of PN12 and PN16 cells was treated with Control or THZ1 at an indicated concentration. **P < 0.01. **G**, **H** The migration of PN12 and PN16 cells with THZ1 treatment following transfected with LIMD1-AS1 plasmid. **P < 0.01 vs. vector, ^##^*P* < 0.01 vs. THZ1. **I**, **J** The invasion of PN12 and PN16 cells with THZ1 treatment following transfected with LIMD1-AS1 plasmid. **P < 0.01 vs. vector, ^##^*P* < 0.01 vs. THZ1.

To address the translational potential of LIMD1-AS1, we prioritized A covalent CDK7 Inhibitor, THZ1, for further investigation based on the expression of LIMD1-AS1 in glioma. We found that LIMD1-AS1 is significantly enriched in patient-derived PN12 and PN16 glioblastoma cells but not MES23, MES27 glioblastoma cells (Fig. 7A). PN12 and PN16 glioblastoma cells migration and invasion were associated with LIMD1-AS1 expression, with THZ1 showing an inhibitory effect (Fig. 7B–F). We next examined whether LIMD1-AS1 was involved in the anti-tumor effect of this THZ1 therapy by treating PN12 and PN16 glioblastoma cells. LIMD1-AS1 partially reversed THZ1-mediated reduction in glioma cell migration (Fig. 7G, H). In addition, LIMD1-AS1 reversed the reduction in invasive glioma cells treated with THZ1 (Fig. 7I, J). These results indicated that glioma cells with high expression of LIMD1-AS1 are susceptible to disturbance by THZ1.

### LIMD1-AS1-regulated malignant phenotype of glioma partially dependents on HSPA5

**Fig. 8:**
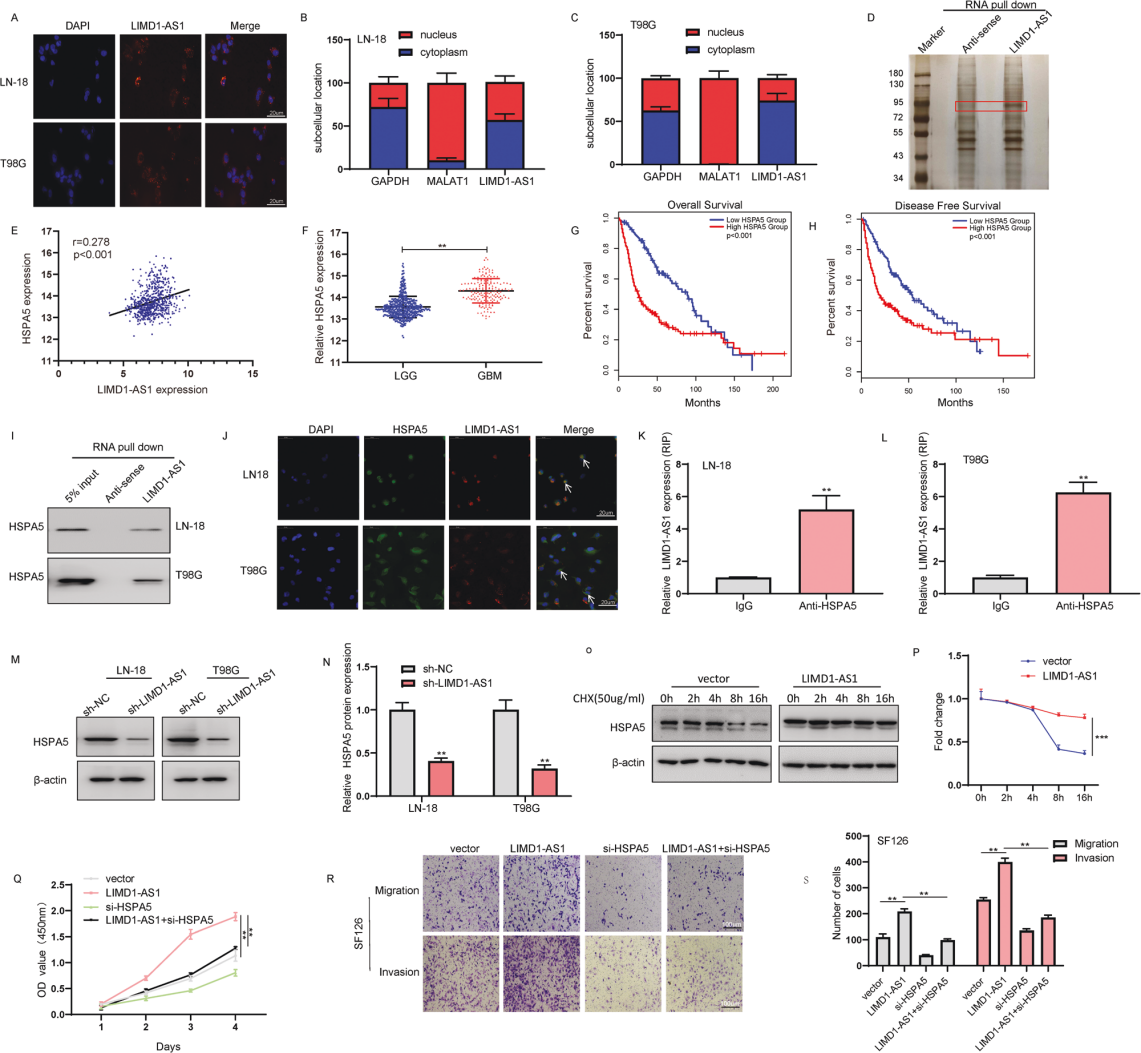
Identification and characterization of LIMD1-AS1 binding to HSPA5. **A** The cellular localization of LIMD1-AS1 in LN-18 and T98G glioma cells was analyzed by RNA-FISH. **B**, **C** The subcellular distribution of LIMD1-AS1 was analyzed via RT-qPCR in LN-18 and T98G glioma cells. MALAT1 and GAPDH were used as nuclear and cytoplasmic markers, respectively. **D** RNA pull-down was performed using sense and antisense LIMD1-AS1, and the retrieved proteins were separated with SDS-PAGE and silver staining. **E** The expression of LIMD1-AS1 is significantly associated with HSPA5 expression in the TCGA database. **F** Expressed HSPA5 between GBM and LGG in TCGA database. **P < 0.01. **G**, **H** Prognostic significance of HSPA5 in gliomas in TCGA database. **I** HSPA5 in LIMD1-AS1 RNA pulldown was analyzed by western blot. **J** The LIMD1-AS1 co-localized with HSPA5 in LN-18 and T98G glioma cells were analyzed by RNA FISH and IF experiments. **K**, **L** RNA immunoprecipitation experiments were performed using an anti-HSPA5 antibody, and specific primers were used to detect LIMD1-AS1. IgG served as the negative control. **P < 0.01. **M**, **N** Relative protein expression of HSPA5 in LN-18 and T98G glioma cells transfected with sh-LIMD1-AS1 or sh-NC. *P < 0.05, **P < 0.01 vs. sh-NC. **O**, **P** The HSPA5 protein level after treatment of the cells with CHX at an indicated time. *p < 0.05. **Q** CCK8 assays were performed to examine the proliferation in SF126 cells transfected with LIMD1-AS1 plasmid or/and si-HSPA5. **P < 0.01. **R**, **S** Transwell assays were performed to observe the migration and invasion of SF126 cells transfected with LIMD1-AS1 plasmid or/and si-HSPA5. **P < 0.01.

Since the molecular mechanism of lncRNA’s effect depends on subcellular localization, we first observe the localization of LIMD1-AS1 in LN-18 and T98G glioma cells. RNA fluorescence in situ hybridization (RNA-FISH) showed that LIMD1-AS1 was distributed mainly in the cytoplasm (Fig. 8A). Nuclear/cytoplasmic fractionation followed by RT-qPCR analysis further confirmed that LIMD1-AS1 was predominantly localized in the cytoplasm, with only weak expression in the nucleus (Fig. 8B, C). Cytoplasmic lncRNAs interact with various protein partners, thus regulating RNA stability, degradation, translation, and splicing of mRNAs [30, 31]. To investigate this phenomenon, we performed RNA pull-down using in vitro synthesized LIMD1-AS1 coupled to biotin, then subjected the precipitants to mass spectrometry (MS) analysis (Fig. 8D). Among these identified proteins, we selected HSPA5 protein base on HSPA5 correlated with LIMD1-AS1, highly expressed in glioma, and associated with overall survival and disease-free survival in glioma patients (Fig. 8E–H, Supplementary Fig. 4A–C). Western blot confirmed that Heat Shock Protein Family A (Hsp70) Member 5 (HSPA5) (also known as GRP78) was co-precipitated with in vitro synthesized LIMD1-AS1 but not with antisense LIMD1-AS1 (Fig. 8I). Moreover, an IF and RNA-FISH assay results showed that LIMD1-AS and HSPA5 were predominantly colocalized in the cytoplasm of LN-18 and T98G glioma cells (Fig. 8J). Subsequently, RT-qPCR analysis following RNA immunoprecipitation assays confirmed the enrichment of LIMD1-AS1 in the complex with HSPA5, compared with IgG control (Fig. 8K, L).

Then, the regulation of LIMD1-AS1 on HSPA5 was evaluated. We found that LIMD1-AS1 knockdown markedly reduced the HSPA5 protein level in the LN-18 and T98G glioma cells (Fig. 8M, N). To further investigate the molecular mechanism by which LIMD1-AS1 up-regulates HSPA5 expression at the posttranscriptional level, the protein synthesis inhibitor cycloheximide (CHX) was used. Overexpression of LIMD1-AS1 in SF126 cells was found to enhance HSPA5 protein stability (Fig. 8O, P). In addition, HSPA5 knockdown partially alleviated the proliferation, migration, and invasion in SF126 glioma cells induced by LIMD1-AS1 overexpression (Fig. 8Q, S). Therefore, our results suggest that LIMD1-AS1 promotes the malignant phenotype of glioma cells by directly binding the HSPA5 protein and enhancing its stability.

### LIMD1-AS1 regulated interferon signaling by HSPA5 in glioma

**Fig. 9:**
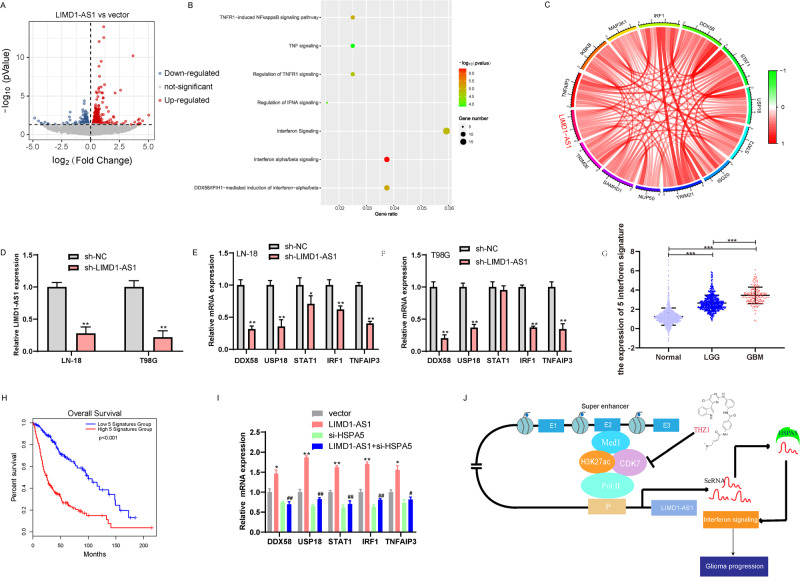
LIMD1-AS1 promotes the expression of an interforen signaling signature by HSPA5. **A** volcano plot showing genes differentially expressed in glioma cells overexpressing LIMD1-AS1 vs. vector cells. **B** Biological functions associated with genes differentially expressed upon LIMD1-AS1 overexpression in glioma cells. **C** Circos plot indicating the correlation of LIMD1-AS1 and interferon signaling in the TCGA database. **D**–**F** Relative mRNA expression of IFN signatures in LN-18 and T98G glioma cells transfected with sh-LIMD1-AS1 or sh-NC. *P < 0.05, **P < 0.01. **G** The IFN signatures expression is shown in normal brain tissues, LGG and GBM in GTEX and TCGA, respectively. ***P < 0.001. **H** Prognostic significance of IFN signatures in gliomas. **I** Relative mRNA expression of IFN signatures in SF126 cells transfected with LIMD1-AS1 plasmid following transfected with si-HSPA5. *P < 0.05, **P < 0.01 vs. vector, ^#^*P* < 0.05, ^##^*P* < 0.01 vs. LIMD1-AS1. **J** A schematic model for the mechanisms of SE driven-LIMD1-AS1 in glioma progression.

To further elucidate the potential signaling pathway of LIMD1-AS1 in promoting glioma progression, we performed RNA-seq analysis to compare the gene expression profiles of LIMD1-AS1 overexpression and control SF126 glioma cells. A total of 596 differential genes were detected after overexpression of LIMD1-AS1 in glioma cells (Fig. 9A). Reactome analysis revealed that these gene sets are mainly related to interferon signals, including Interferon alpha/beta signaling, DDX58/IFIH1-mediated induction of interferon-alpha/beta, Interferon Signaling (Fig. 9B). In addition, we confirmed that LIMD1-AS1 is positively associated with interferon signaling in the TCGA and CGGA database (Supplementary Fig. 5A, B). Next, we focus on the top-ranked differential genes in the three gene sets, and significantly correlated with the expression of LIMD1-AS1 in the TCGA and CGGA database (Fig. 9C, Supplementary Fig. 6A–J), indicating LIMD1-AS1 and interferon signaling were usually co-expressed in glioma. These genes of interferon signaling, including DDX58, USP18, STAT1, IRF1, and TNFAIP3 (IFN signatures), are for further analysis. We found that the mRNA expression of IFN signatures was reduced in LIMD1-AS1 knockdown glioma cells (Fig. 9D–F). IFN signatures mRNA levels were significantly upregulated in glioma samples compared with normal brain tissue, and upregulation of IFN signatures was associated with glioma malignancy (Fig. 9G). The high IFN signatures are significantly associated with poor survival in patients with glioma (Fig. 9H, Supplementary Fig. 7A–J). Next, we explore whether HSPA5 was involved in the downregulation of the interferon signaling caused by LIMD1-AS1 knockdown. We transfected HSPA5 siRNA into LIMD1-AS1-overexpressed SF126 glioma cells and found that knockdown of HSPA5 expression significantly reduced LIMD1-AS1-induced increase IFN signatures mRNA levels in SF126 glioma cells (Fig. 9I). Therefore, these data indicate that LIMD1-AS1’s regulation of interferon signaling is at least partially dependent on HSPA5 in gliomas.

## Discussion

Gliomas are the most common form of central nervous system malignant tumors with high recurrence, poor prognosis, and mortality rate [32]. Hence, it is desirable and urgently needed to identify the potential molecular mechanisms underlying glioma progression and provide evidence for novel therapeutic targets. In this paper, we identified LIMD1-AS1 as a novel oncogenic lncRNA that could promote the growth and invasion of glioma cells. Mechanistically, the SE complex CDK7 recruits MED1 to the SE region of LIMD1-AS1, which activates the transcription of LIMD1-AS1. Finally, LIMD1-AS1 directly interacts with HSPA5 and further specifies the transcriptional activation pattern on interferon signaling in glioma (Fig. 9J).

SEs are defined as clusters of enhancers in close genomic proximity that drives the expression of genes that are cell-type specific, associated with essential cell identity genes, and linked to many biological processes [16]. There is accumulating body of evidence that SEs are involved in the regulation of tumor development and maintaining a malignant phenotype [33]. Recent studies have shown that protein-coding genes driven by SEs have been found in several tumors, including glioma [19, 34–36]. However, non-coding RNAs, especially lncRNAs, driven by SEs in gliomas are still largely unknown. At first, the identification of SE was mainly through ChIP-seq analysis using a combination of active enhancer marks (H3K27ac, H3K4me1), the Mediator complex (MED1), and Transcription factors (TFs) profiles [37, 38]. Young et al. reported that bioinformatics algorithms via ROSE software locate genomic proximity for grouping elements to assign SE to a putative target gene [39]. Currently, we use ROSE’s combination with the activity of molecular H3K27ac and MED1 as analyzed by ChIP-Seq to identify SEs. This paper identified a SE associated with LIMD1-AS1 in GBM cells with integrative ChIP-seq and RNA-seq analysis. Further in vitro and in vivo, functional studies have provided striking evidence that LIMD1-AS1 promotes cell proliferation, migratory and invasive capacities in glioma. Moreover, high expression of LIMD1-AS1 is correlated with the poor prognosis of glioma patients. These findings indicated that LIMD1-AS1 exhibited strong oncogenic potential in glioma.

Super enhancer-driven transcriptional activation is closely related to the malignant biological behavior of tumor cells [40]. Based on the fact that tumor cells may be highly dependent on transcriptional programs, this may provide the potential for tumor therapeutic intervention. As the core transcription complex architecture, SEs-complex is involved in the transcription process of oncogenes [41, 42]. Thus, transcriptional inhibition of oncogenes by targeting the SEs-complex has become an attractive strategy for cancer therapy. Although unique to cancer cells, the same oncogene can form a different SE architecture in various tumor types [16]. Since SE components are common in different tumor cells, we can directly inhibit common SEs components and thus hamper the oncogene from becoming addicted to SEs [43–45]. Here, we found that SE-complex components, such as CDK7, significantly correlated with the expression of LIMD1-AS1. Inhibition of CDK7 reduced the transcriptional activity of the LIMD1-AS1 promoter and enhancer, leading to reduced expression of LIMD1-AS1.

CDK7 is a ubiquitously expressed CDK-activating kinase (CAK) which activates other CDKs involved in cell-cycle control, and acts as a component of the general transcription factor TFIIH, which phosphorylates the largest subunit of Pol II [46]. Cancer cells are addicted to transcription driven by SEs and thus highly dependent on CDK7 activity, as exemplified by the identification of a so-called “Achilles cluster” of genes in MYCN amplified glioblastoma, triple-negative breast cancer [16], and pancreatic cancer [18, 47, 48]. CDK7, a component of TFIIH, then phosphorylates the CTD, which disrupts the CTD interaction with the Mediator complex, including MED1, and is presumably necessary to release RNA polymerase II from promoter-proximal pausing [28]. In this study, we deciphered that CDK7 phosphorylates the transcriptional co-activator MED1. However, it is necessary to observe whether a hypersensitive subset can be identified through phospho-MED1 levels in glioma hypersensitive to CDK7 inhibition. Previous studies have shown that MED1 is phosphorylated by CDK7 at residue T1457 or/and T1032, and reinforces the mediator complex recruitment to chromatin to activate gene transcription [49]. Thus, we investigated whether CDK7 affected the LIMD1-AS1 enhancer activity in a manner dependent on MED1. Our results confirmed that inhibition of CDK7 attenuates MED1 recruitment to the super-enhancer of LIMD1-AS1 and then decreases the expression of LIMD1-AS1 in glioma cells. These findings indicate that CDK7 enhances the transcriptional activation of LIMD1-AS1, at least in part through MED1 in glioma. However, how does CDK7 phosphorylates MED1? How does CDK7 identify the phosphorylation site of MED1? In future work, we will further address the above questions.

Targeting SE-associated oncogenic transcription programs by a small-molecule CDK7 inhibitor shows powerful antineoplastic properties against tumor cells [45]. As a highly specific covalent inhibitor of CDK7, THZ1 represses transcription by decreasing CDK7-dependent Pol II CTD phosphorylation [50, 51]. Recently, one published paper reported that THZ1 treatment resulted in global gene transcription repression in glioma cells, preferentially targeting the SE-associated genes, which eventually destroy glioma cells’ viability [19]. Here, treatment with THZ1 also substantially hampered LIMD1-AS1 promoter and enhancer activity, and then inhibited the transcription of LIMD1-AS1. To explore the translational potential of THZ1, we tested its effects on patient-derived glioma cells. We found that glioma patients with high expression of LIMD1-AS1 may be susceptible to disturbance by THZ1. Currently, SY1365, a covalent CDK7 selective inhibitor, is used in clinical trials for advanced breast and ovarian cancer (NCT03134638). Our findings provide further evidence that a covalent CDK7 inhibitor may benefit glioma patients with high expression of LIMD1-AS1. However, more works should elucidate the mechanism of SEs-driven lncRNA transcription addiction and use these new targets to block transcription to treat tumors. Recent studies have shown that targeting lncRNA could be a promising treatment for glioma. Specifically, the treatment of lncRNA is mainly through correcting pathological changes in lncRNA expression, including reactivation of endogenous lncRNA and functional blocking or reduction of lncRNA expression [52]. Diverse methods, including CRISPR/Cas9-mediated genome editing, anti-sense oligonucleotides (ASO), ribozymes, aptamers, and small molecules interfering with lncRNA biogenesis [53, 54], can be used to alter LIMD1-AS1 expression. However, many challenges remain in delivering these therapies in an organ-, tissue- or cell-type-specific manner to avoid off-target effects.

LncRNAs exert their roles in all cell functions operating via multiple molecular mechanisms. A few better-characterized lncRNAs, such as MALAT1, XIST, and HOTAIR, are chromatin-associated lncRNAs. They regulate transcription factor activation, RNA polyadenylation, RNA transport, and chromatin modifications [55–58]. However, the exact functions of cytoplasmic lncRNAs, particularly their potential functions in the regulation of signaling pathways, remain poorly understood. FISH experiments showed that LIMD1-AS1 is primarily located in the cytoplasm but is also a certain proportion in the nucleus of glioma cells, suggesting that LIMD1-AS1 has a complex regulatory mechanism in glioma. Although lncRNA regulates interferon signaling has been reported in tumors, here we found that LIMD1-AS1 is positively associated with interferon signaling in glioma, whereas knockdown of LIMD1-AS1 markedly inhibits interferon signaling in glioma cells. Recent reports suggest that interferon signaling is a significant driver of brain tumor progression and cellular heterogeneity, contributing to the immune evasion of glioma cells [59]. However, whether and how these pathways are regulated by lncRNA remains largely undeciphered. Moreover, we found that a small part of LIMD1-AS1 is distributed in the nucleus, suggesting that LIMD1-AS1 has potential additional nuclear functions.

The network of lncRNAs and their interacting partners play a vital role in tumor progression [60]. LncRNAs usually exert their effects by RNA-protein interactions [61]. To further explain the in-depth mechanisms of LIMD1-AS1-induced interferon signaling activation in glioma, we investigated proteins that could specifically bind LIMD1-AS1 in glioma. Presently, we demonstrated that LIMD1-AS1 could directly bind to HSPA5 in the cytoplasm and positively regulate its expression. HSPA5 contributes to cancer development and progression as an oncogene [62]. Its overexpression is correlated with poor prognosis in diverse malignancies, including gliomas [63]. UPR upregulation by HSPA5 suppression alleviated interferon-mediated liver injury [64]. Next, we explore whether HSPA5 is involved in LIMD1-AS1-induced interferon signaling activation. Our findings verified that LIMD1-AS1 promoted interferon signaling activation in an HSPA5-dependent manner. Therefore, our findings built a bridge between the epigenetic networks of lncRNAs and interferon signaling. Further studies are warranted to determine whether the LIMD1-AS1-HSPA5-interferon axis contributes to the development of chronic inflammatory diseases and other cancers. VAP peptides, exhibiting high binding affinity in vitro to HSPA5 overexpressed on glioma, was a flexible and multifunctional peptide to mediate glioma targeting [65]. However, whether VAP peptide can better treat gliomas with high expression of LIMD1-AS1 needs further investigation.

In summary, our results provide novel evidence that epigenetic modifications of LIMD1-AS1 are mediated by CDK7-activated interferon pathways by directly binding HSPA5, which might contribute to glioma progression. These findings will increase our knowledge of the biological basis of glioma progression and might allow the development of novel therapeutic drugs for patients with glioma.

## Materials and methods

### Tissue samples

Human normal brain tissue (*n* = 12) and glioma tissue (*n* = 43) were collected from patients at the Department of Neurosurgery of the Second Affiliated Hospital of Anhui Medical University. The clinical features and information on glioma are in Supplementary Table 1. This study was approved by the Research Ethics Committee of The Second Affiliated Hospital of Anhui Medical University, and written informed consent was obtained from all patients.

### Cell culture and treatment

Human normal human astrocytes (NHAs) were obtained from Sun Yat-Sen University, glioma cells (H4 and SF126) were obtained from the Cell Bank of the Chinese Academy of Sciences (Shanghai, China), and (U251, LN-18, T98G, and SW1088) were purchased from American Type Culture Collection (Manassas, VA), and mycoplasma detection and STR cell identification were carried out. MES23, MES27, PN12, and PN16 cells were isolated from patient-derived glioblastoma specimens as we previously reported [66]. All siRNAs, targeting LIMD1-AS1, CDK7, HSPA5, and negative control, were purchased from Genepharma (Shanghai, China). The full-length LIMD1-AS1 cDNA was cloned into the pcDNA3.1 vector (Invitrogen). All target siRNA sequences are shown in Supplementary Table 2. All the cells were incubated in DMEM medium (Invitrogen, Carlsbad, CA, USA) supplemented with 10% fetal bovine serum (HyClone, Logan, UT, USA) and were maintained in a humidified incubator at 37 °C with 5% CO_2_. Where indicated, glioma cells were treated with THZ1 (MCE, Saint Louis, MO, USA) for 48 h, as shown in the article.

### In vitro functional assays

#### Cell proliferation assay

Glioma cells were seeded in 96-well plate with a concentration of 3000 cells per well, and cultured at 37 °C. The cell number was measured by the Beckman Z1 particle counter (Beckman Coulter). The absorbance in each well was measured at 490 nm with a microplate reader and cell proliferation curves were plotted against time.

#### Colony formation assay

Glioma cells (5 × 10^2^) were seeded into wells of a six-well plate to allow colony formation. After ten days, the cells were fixed with 10% formaldehyde for 10 min, washed with PBS. Cell colonies formed were stained with Giemsa for 15 min.

#### In vitro cell migration and invasion assay

The Transwell cell migration and invasion assays were performed using a 24-well plate with 8-μm-pore size chamber inserts (Corning, USA). The invasion assay was performed by the same procedure as the migration assay, except that the membrane was coated with Matrigel to form a matrix barrier. For the migration assay and invasion assay, 1 × 10^5^ cells were seeded in the upper chamber (BD Bioscience) in culture medium with 1% FBS, and 500 µl of culture medium with 10% FBS was added in each lower chamber, and then they were all incubated for 24 or 48 h at 37 °C. After incubation, the bottom of transwell inserts was fixed with cold methanol and stained with 0.5% crystal violet. Subsequently, migrated, and invaded cells were counted in five randomly selected fields under a microscope.

### RNA-FISH and subcellular fractionation

The RNA FISH probe mix for LIMD1-AS1 was procured from RiboBio (Guangzhou, China) and employed under the supplier’s instruction. The nuclear and cytoplasmic fractions of glioma cell lines were isolated by the PARIS™ Kit Protein and RNA Isolation System (Invitrogen, USA) according to the manufacturer’s protocol. MALAT1 and GAPDH were used as the nuclear and cytoplasmic controls, respectively.

### Dual-luciferase reporter assays

A Dual-Luciferase Reporter Assay System (Promega, Madison, WI, USA) was adopted to determine the firefly luciferase activity, with Renilla luciferase serving as a transfection control. All experiments were performed in triplicate.

### RNA pull-down + MS/WB

RNA pull-down is performed as we previously described [22]. Biotin-labeled LIMD1-AS1 were obtained by transcription in vitro (T7 RNA Polymerase, 10×Transcription buffer, Roche), labeled with biotin RNA labeling mix (Roche), and then incubated with extracts separated from glioma cells. Pulldown components were separated by SDS-PAGE and silver staining. Finally, differential bands were analyzed by mass spectrometry (MS) or western blot.

### Co-immunoprecipitation (Co-IP) assay

Co-immunoprecipitation (Co-IP) was used to detect the interaction between CDK7 (Cell Signaling Technology) and MED1 (Bethyl), and antibody-protein complex capture with protein A/G agarose (Bimake, B23201) at 4 °C for 24 h. The obtained immune complexes were separated by SDS-PAGE and subjected to western blot analysis using anti-CDK7 or anti-MED1.

### Chromatin immunoprecipitation (ChIP)

ChIP assays were performed with glioma cells using the SimpleChIP Plus Sonication Chromatin IP kit (Cell Signaling Technology, USA) according to the manufacturer’s protocol. Briefly, Chromatin was immunoprecipitated with anti-H3K27ac (Cell Signaling Technology) or anti-IgG as a negative control. ChIP DNA was extracted and analyzed by RT-qPCR. Primer sequences are listed in Supplementary Table 3.

### Immunocytochemistry, immunofluorescence, western blot, and RT-qPCR

Immunocytochemistry, immunofluorescence, western blot, and RT-qPCR were performed according to standard protocols [22]. Primer sequences are listed in Supplementary Table 4. The following antibodies were listed in Supplementary Table 5.

### RNA immunoprecipitation (RIP)

The Magna RNA-binding protein immunoprecipitation kit (Thermo Fisher Scientific, USA) was employed to conduct the RIP assay. Mouse IgG was used as a negative control. The relative enrichment in the RIP-qPCR assay was normalized by input.

### CRISPR/dCas9-KRAB interference

Expressing dCas9-KRAB glioma cells was first established by infection with lentivirus produced from the pHAGE-EF1-dCas9-KRAB plasmid (50919, Addgene) [67], infected cells were subject to Puromycin selection (2 µg/ml) (ThermoFisher Scientific). For CRISPRi assay, individual sgRNAs targeting the super-enhancer region of LIMD1-AS1 were cloned into the lentiviral plasmid and infected into dCas9-KRAB expressing glioma cells. RNA was extracted and RT-qPCR was performed. The gRNA sequences for the LIMD1-AS1 enhancers were as listed in Supplementary Table 6.

### Tumor formation study in vivo

All experimental protocols involving mice were in strict adherence to the guidelines of the Animal Care and Use Committee of Anhui Medical University. Six-week-old BALB/c female nude mice were purchased from Beijing HFK Bioscience Co.,LTD. For subcutaneous xenograft models, mice were randomly divided into two experimental groups (*n* = 6), a total of 5 × 10^6^ cells in 100 μl of PBS were injected subcutaneously into 6-week-old BALB/c-nude mice. Tumor growth was assessed with calipers once a week, and tumor volume was calculated according to the formula: *V* = (length × width^2^)/2. The mice will not be included for statistical analysis once the mice dead or the tumor diameter is >2 cm. The animal experiments in this study were approved and reviewed by the Animal Research Committee of Anhui Medical University.

### RNA-seq analysis

Total RNA was separately extracted from glioma cells transfected by LIMD1-AS1 plasmid and sent to the company for RNA-seq. Following sequencing, the sequencing reads were trimmed to remove adaptor sequence and low-quality bases. The fastp software was used to remove the reads that contained adaptor contamination, low-quality bases, and undetermined bases with default parameters. The sequencing reads were aligned and mapped to the UCSC human hg38 genome assembly with HISTAT2. The generated BAM files containing the results of the alignment were sorted, and raw read counts for each gene were calculated using the featureCounts function. Gene expression in reads per kilobase per million mapped reads (RPKM) was calculated using in-house scripts.

### Correlation between expression/methylation and expression/copy number

Data on gliomas RNA-seq data, copy-number data, and 450k methylation data with clinicopathological features were obtained from UCSC Xena (https://xena.ucsc.edu). Correlated methylation, copy number, and expression data were taken from the same samples, and the Pearson correlation coefficient and the p-value were calculated.

### ChIP-seq analysis

Raw fastq files (GSE36354) were downloaded from the European Nucleotide Archive using Aspera, and then map reads to hg38 genome with bowtie2 and sort them by samtools. Genome coverage bigWig files were generated by converting BAM files to bedGraph format using genomeCoverageBed and the peak calling by using macs14. Super enhancers were identified using the ROSE algorithm with the exclusion of peaks within +/−2.5 kb of TSS and a stitch distance of 12.5 kb and visualize them by using IGV.

### Statistical analysis

Assays were performed in triplicate and results are expressed as mean ± SD of three independent experiments. Student’s *t*-test or one-way ANOVA was used for statistical analysis. Pearson’s correlation was used to investigate the relationship between the variables. Statistical significance was defined as *P* < 0.05.

## Supplementary information

Original Data

Supplement

Checklist

Supp Figure 1

Supp Figure 2

Supp Figure 3

Supp Figure 4

Supp Figure 5

Supp Figure 6

Supp Figure 7

## Data Availability

Data supporting this study are available from the corresponding authors upon reasonable request. RNA-seq data associated with this study has been submitted to GEO database and can be accessed with the ID: PRJNA953138.
