# Supplementary figures and images for: Super-enhancer-driven lncRNA LIMD1-AS1 activated by CDK7 promotes glioma progression
Source: Cell Death Dis. 2023 Jun 29;14(6):383. doi: 10.1038/s41419-023-05892-z (PMC10310775; doi:10.1038/s41419-023-05892-z)

Fig.3O

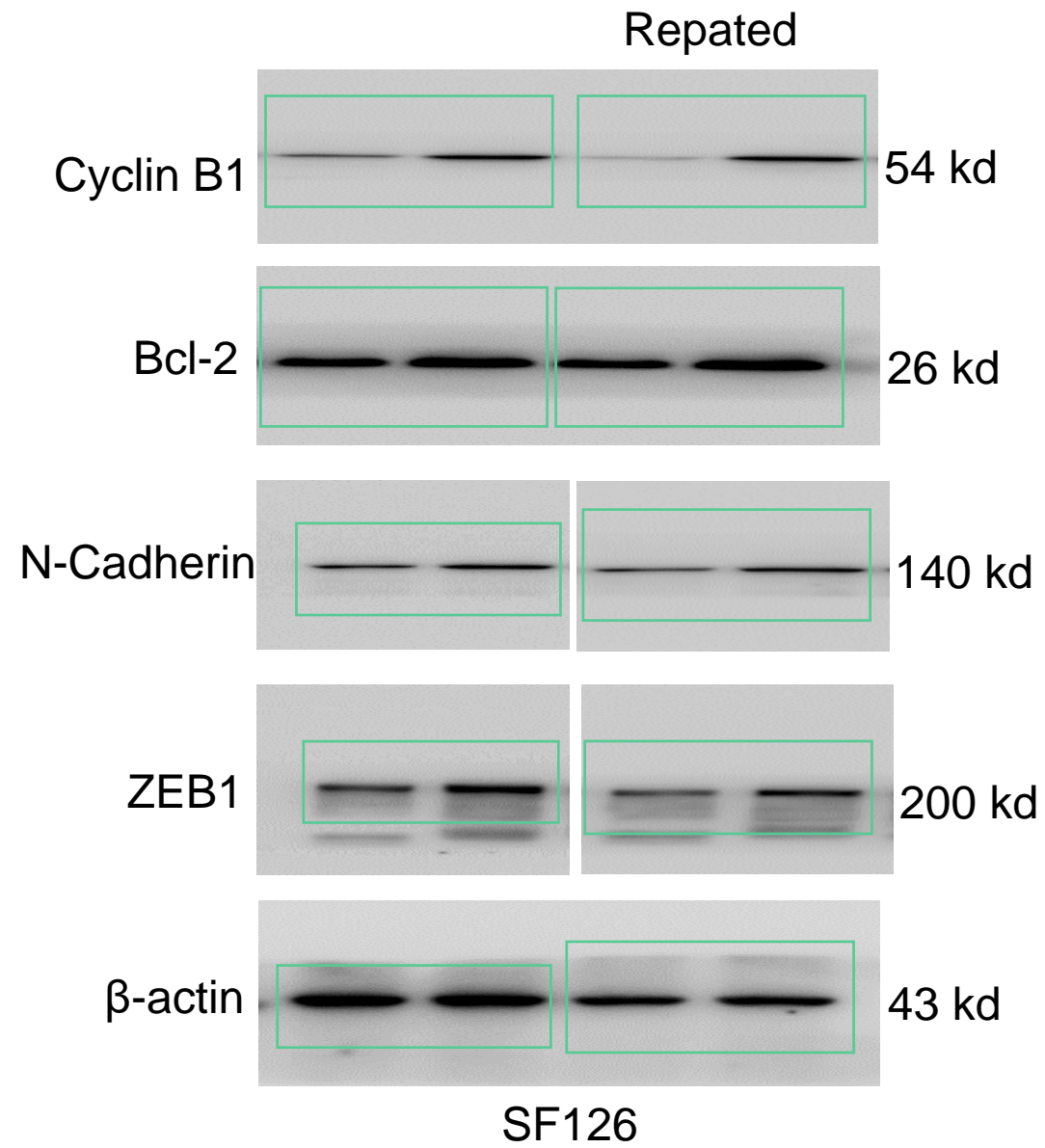

Fig.6 I

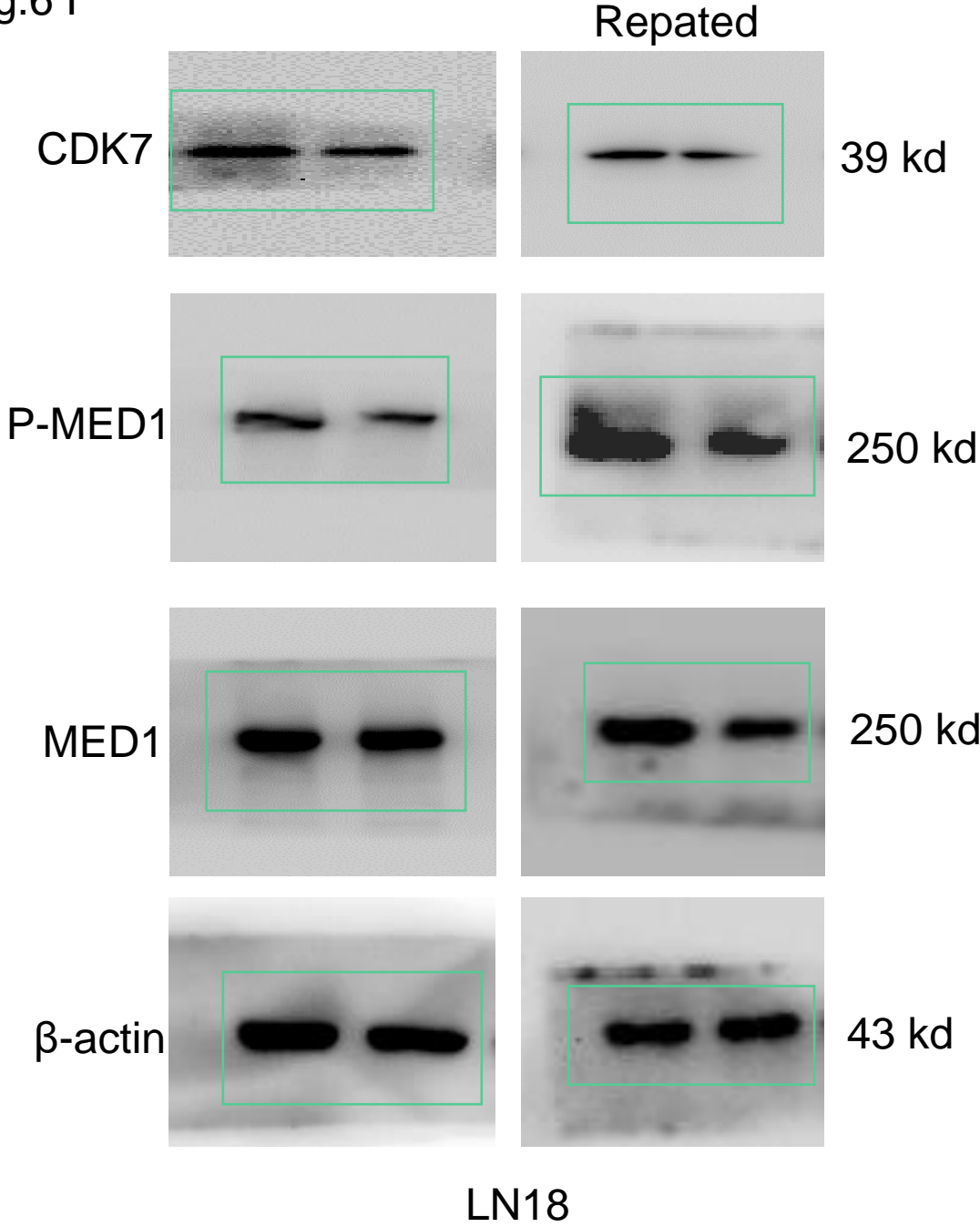

Fig.6K

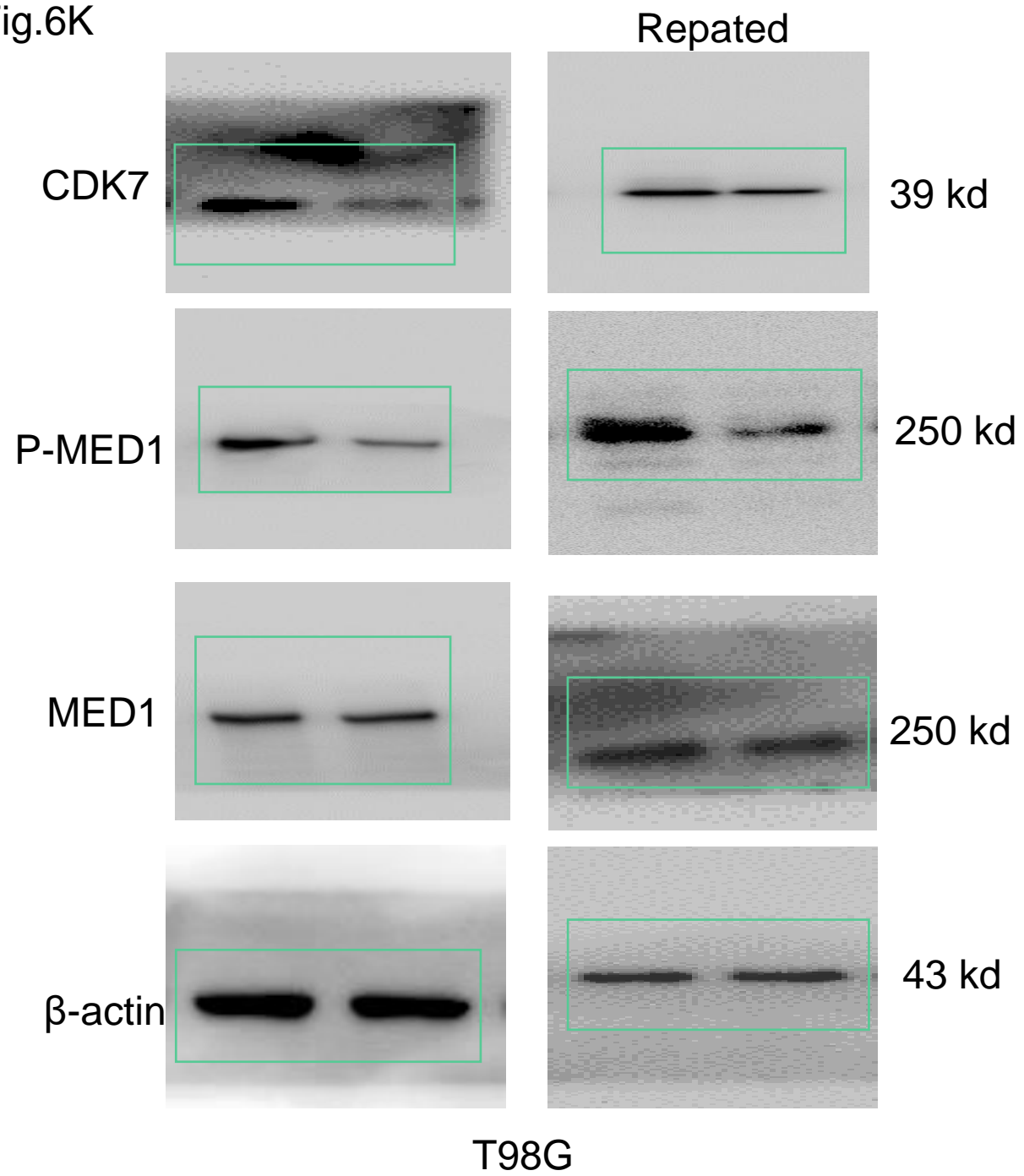

Fig.6 O

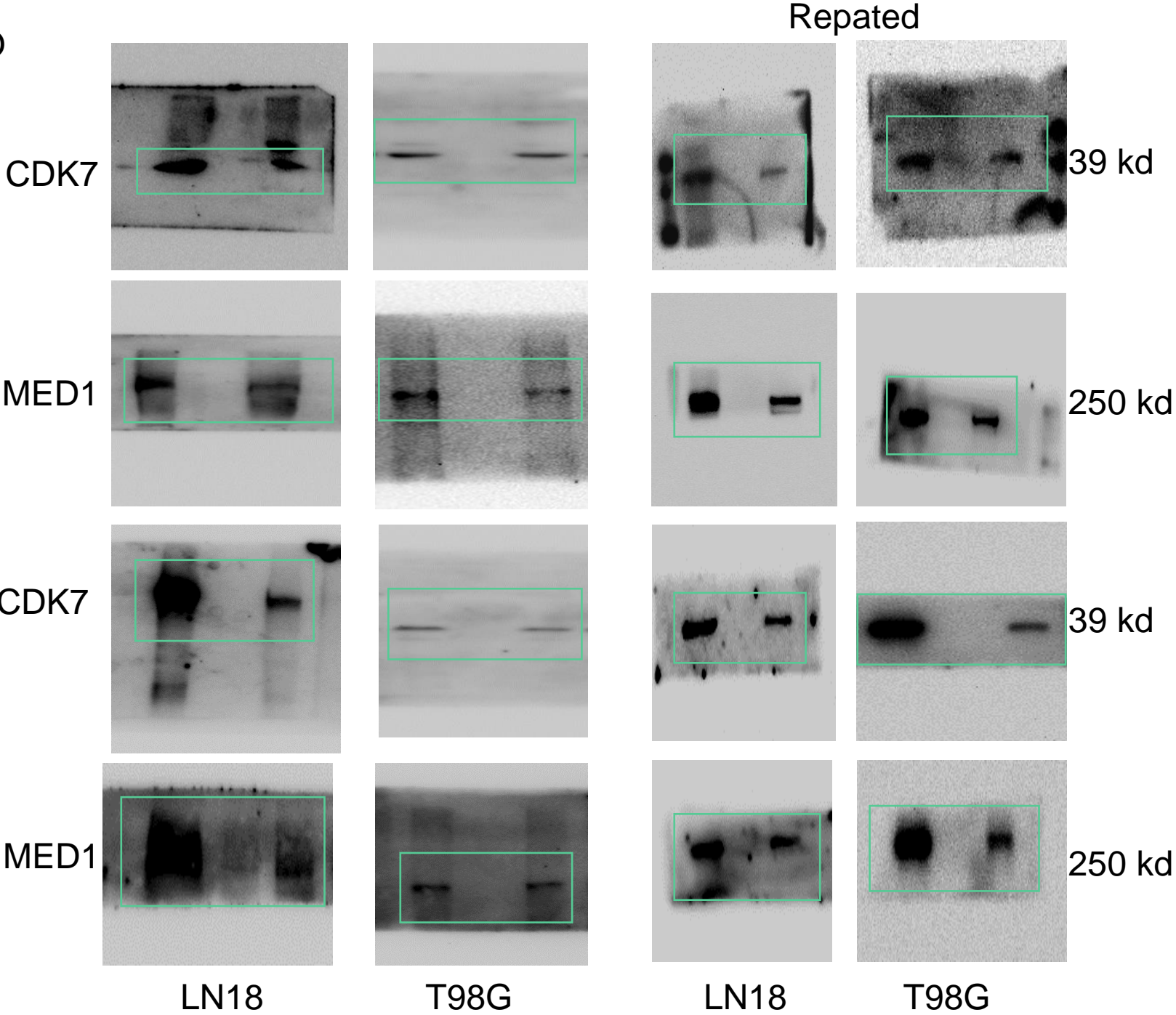

Fig.8M

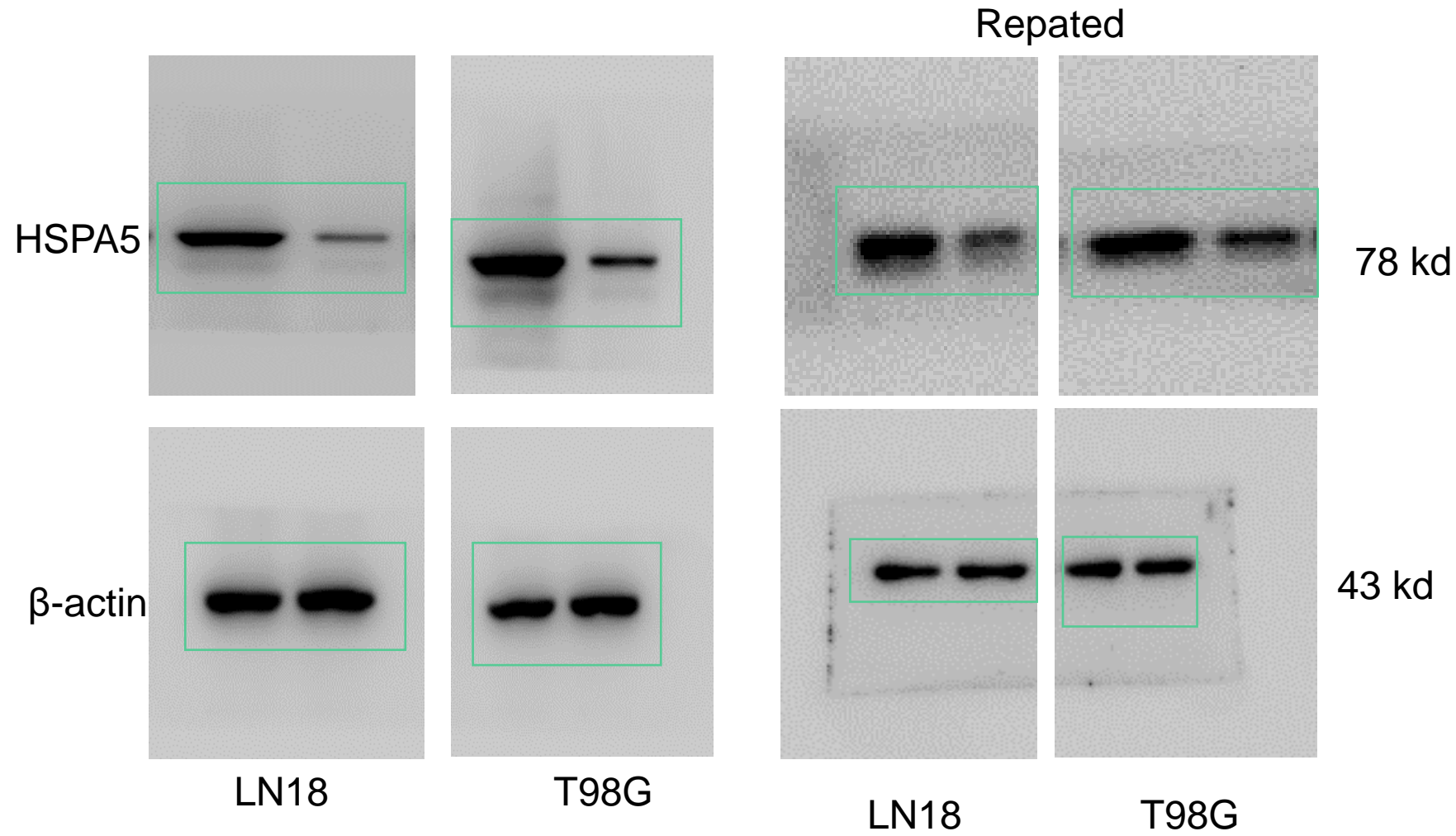

Fig.8I

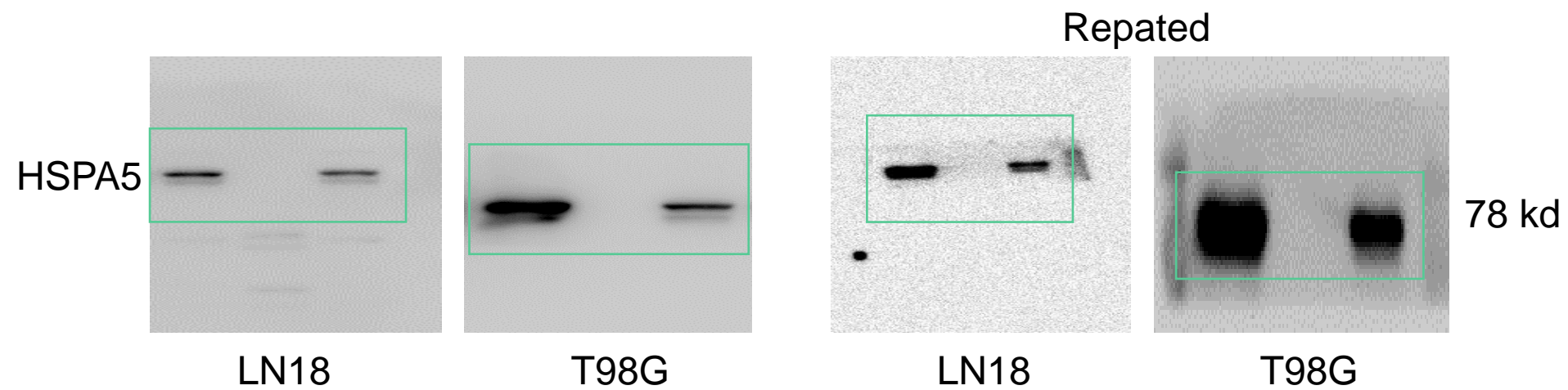

Fig.8O

SF126

HSPA5

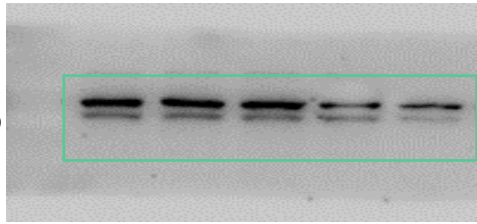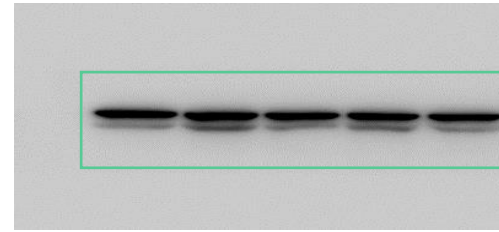

78 kd

$\beta$ -actin

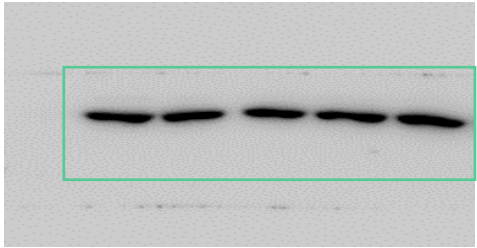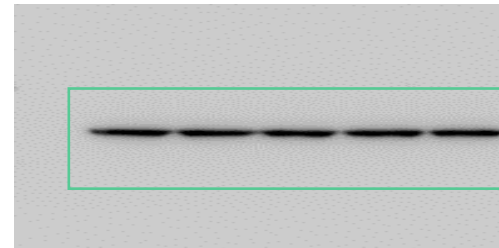

43 kd

Repatd

HSPA5

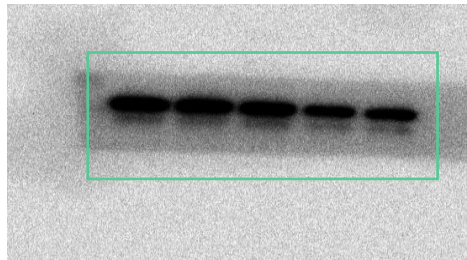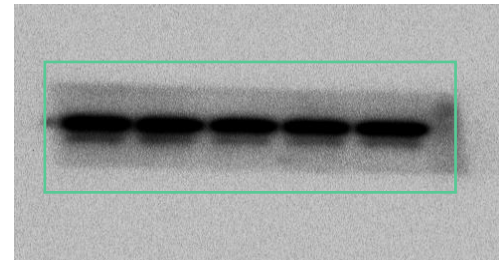

78 kd

$\beta$ -actin

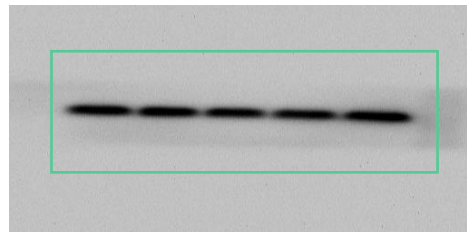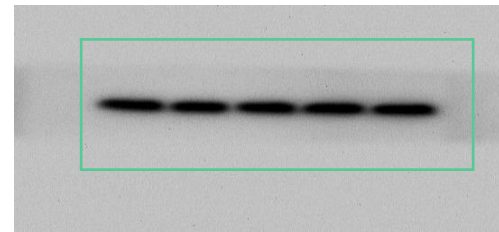

43 kd

Supplement: Supplementary file 1 — Original Data [file 41419_2023_5892_MOESM1_ESM.pdf]

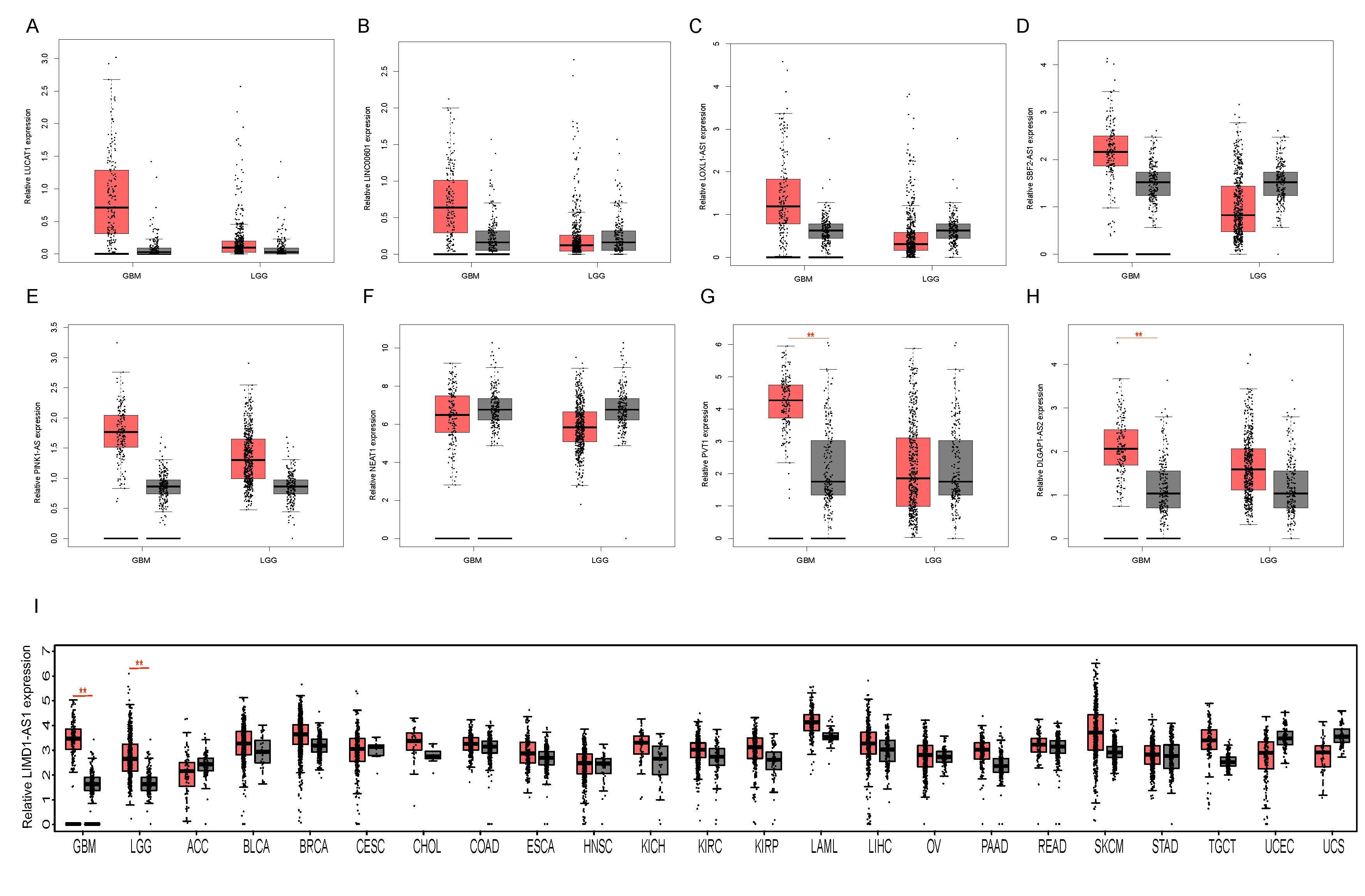

Supplement: Supplementary file 4 — Supp Figure 1 [file 41419_2023_5892_MOESM4_ESM.tif]

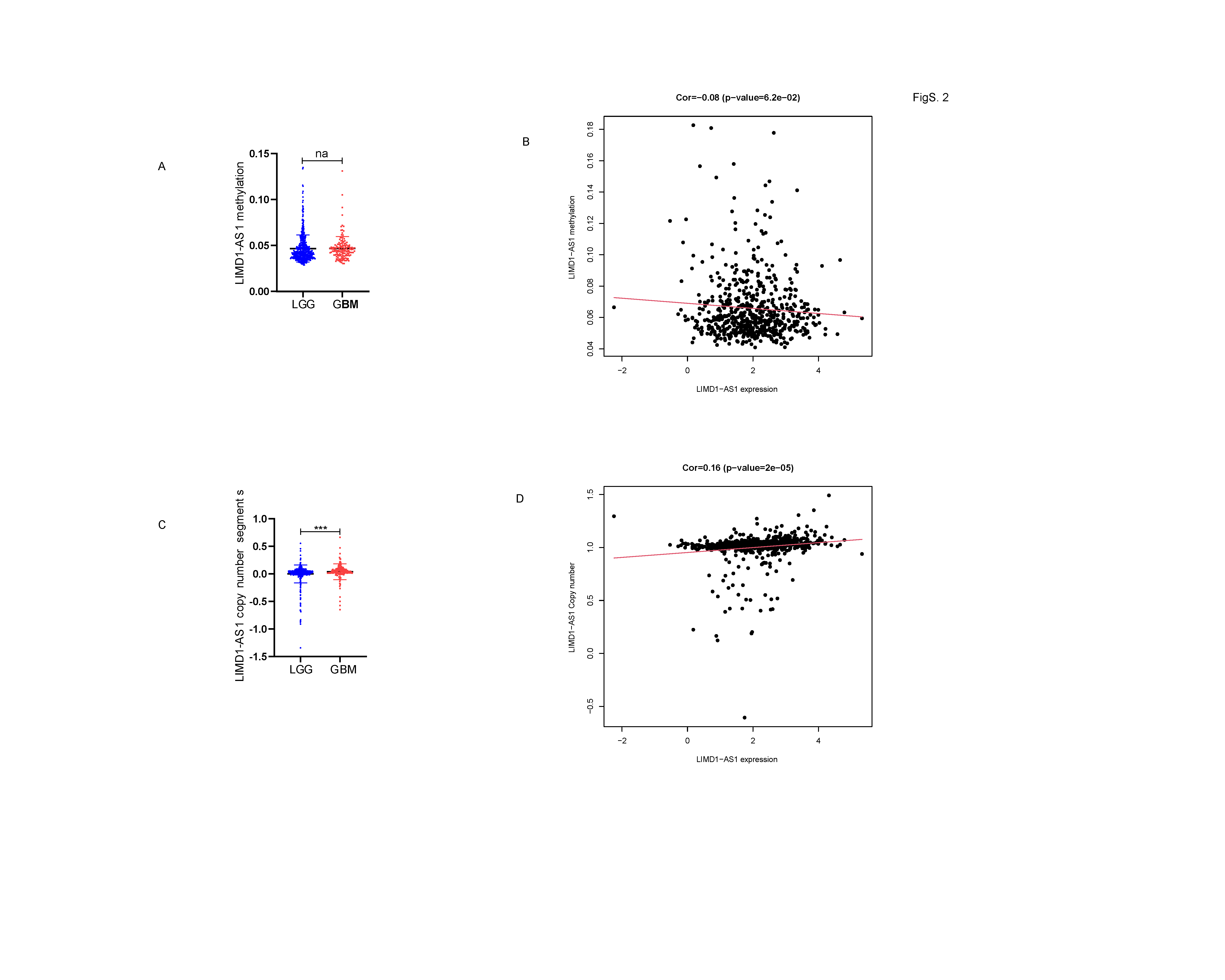

Supplement: Supplementary file 5 — Supp Figure 2 [file 41419_2023_5892_MOESM5_ESM.tif]

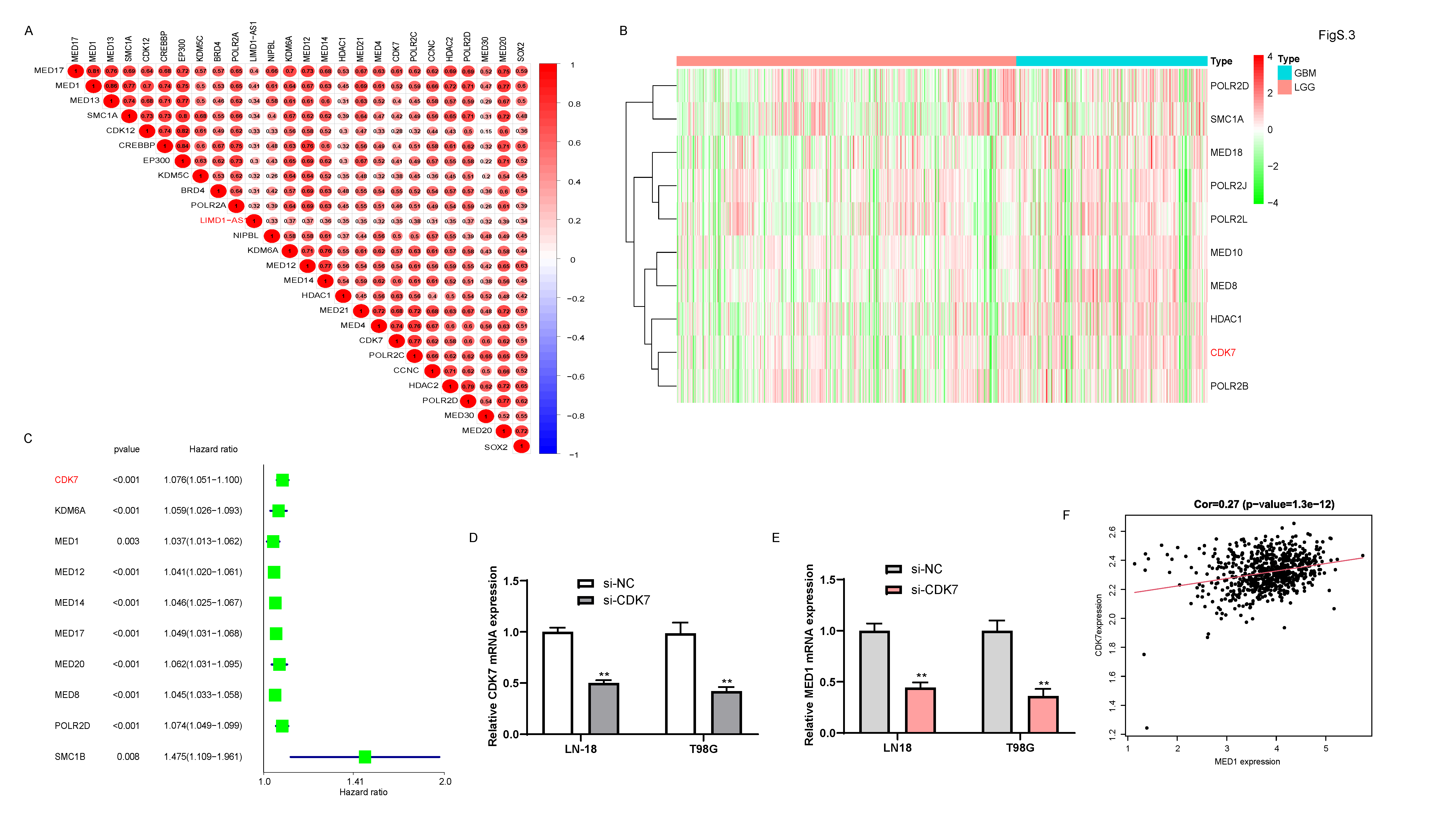

Supplement: Supplementary file 6 — Supp Figure 3 [file 41419_2023_5892_MOESM6_ESM.tif]

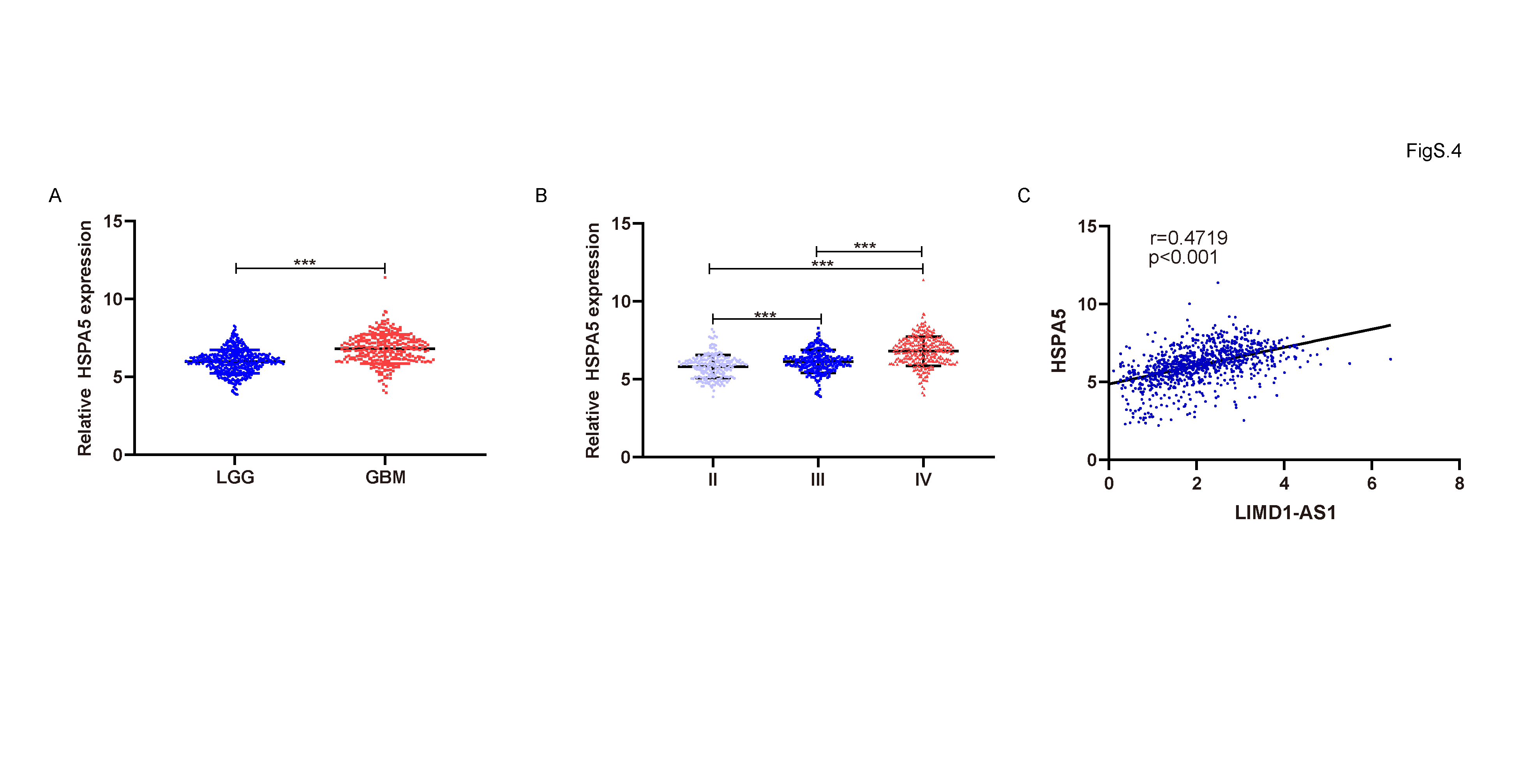

Supplement: Supplementary file 7 — Supp Figure 4 [file 41419_2023_5892_MOESM7_ESM.tif]

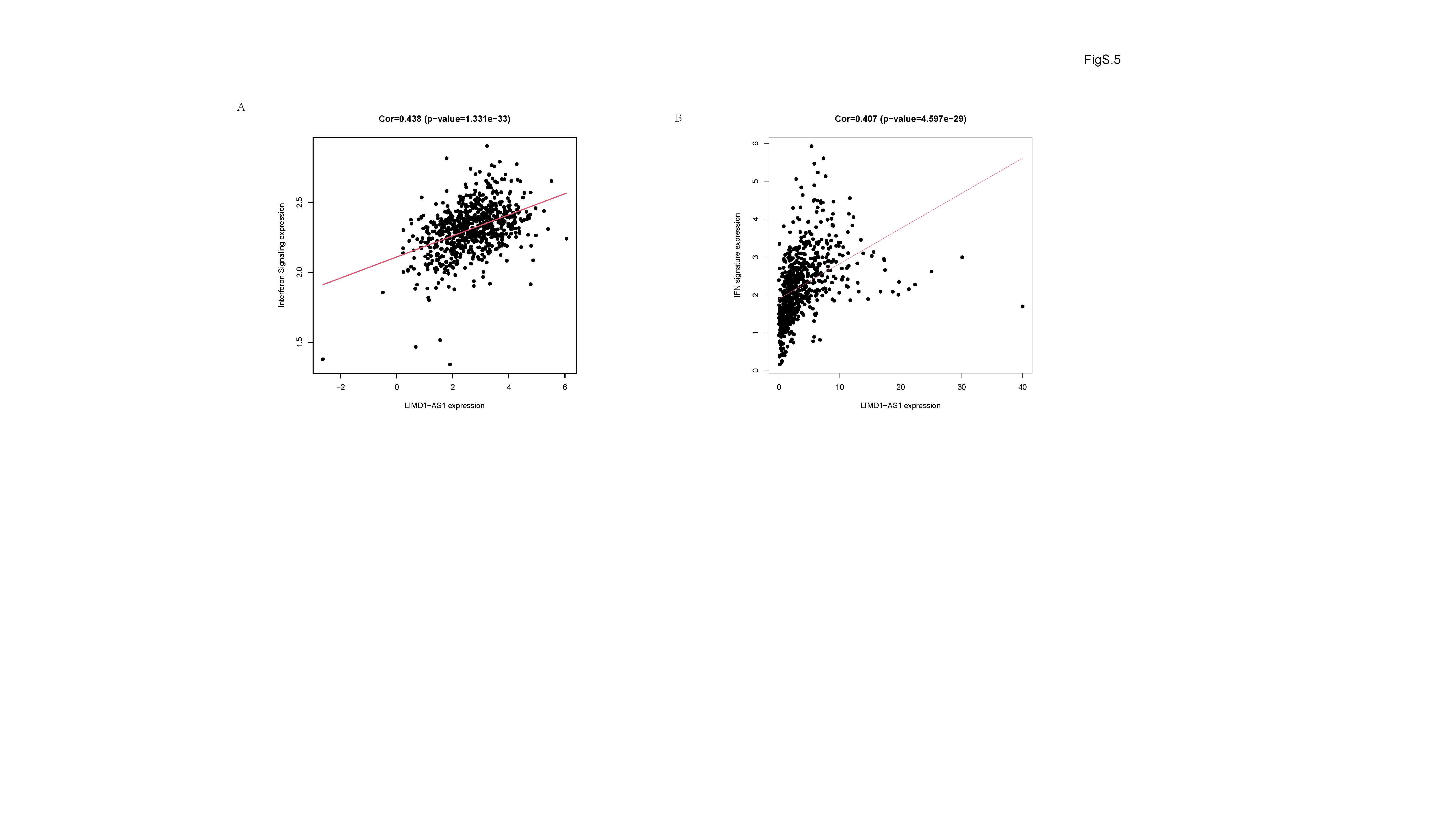

Supplement: Supplementary file 8 — Supp Figure 5 [file 41419_2023_5892_MOESM8_ESM.tif]

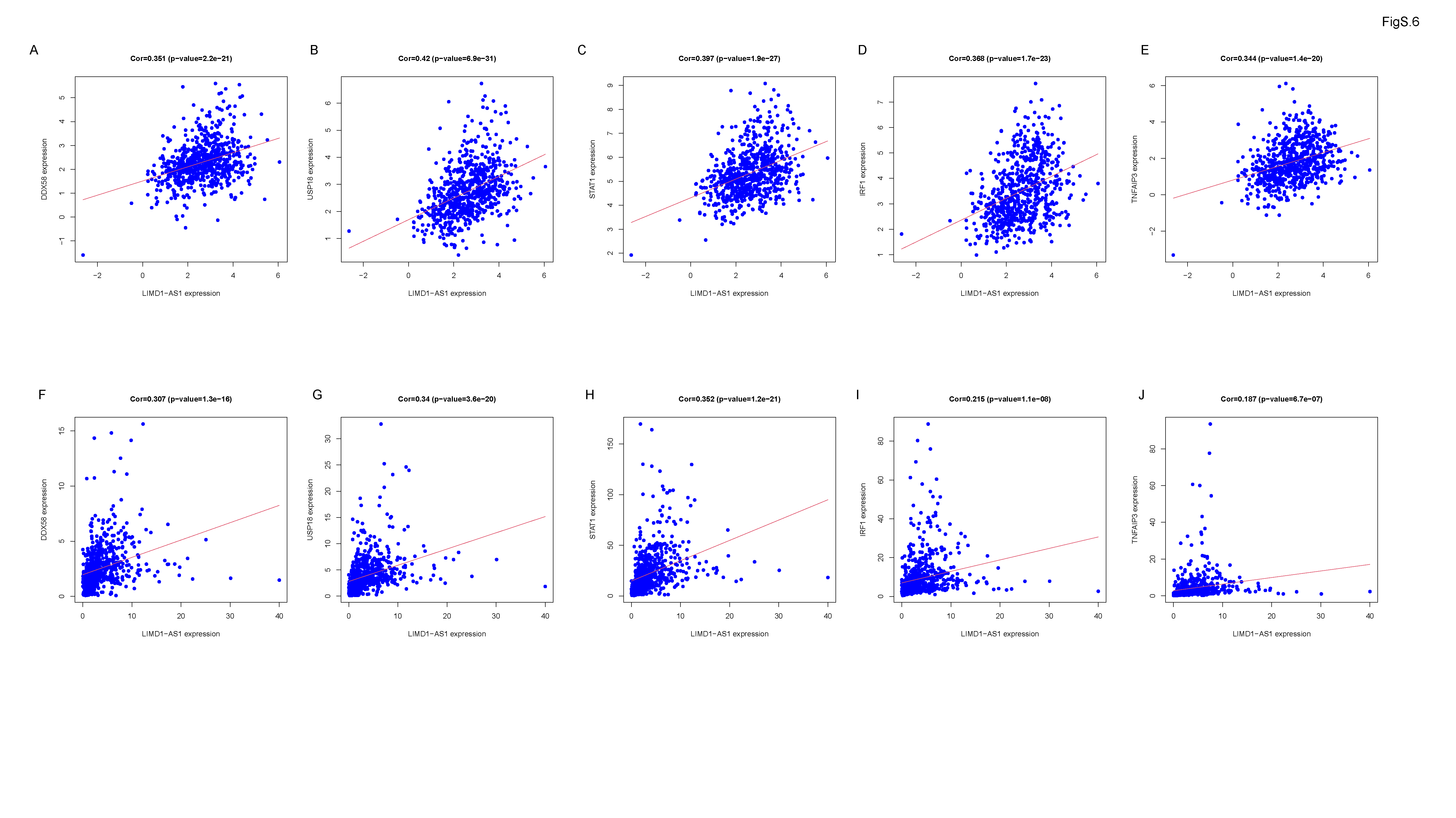

Supplement: Supplementary file 9 — Supp Figure 6 [file 41419_2023_5892_MOESM9_ESM.tif]

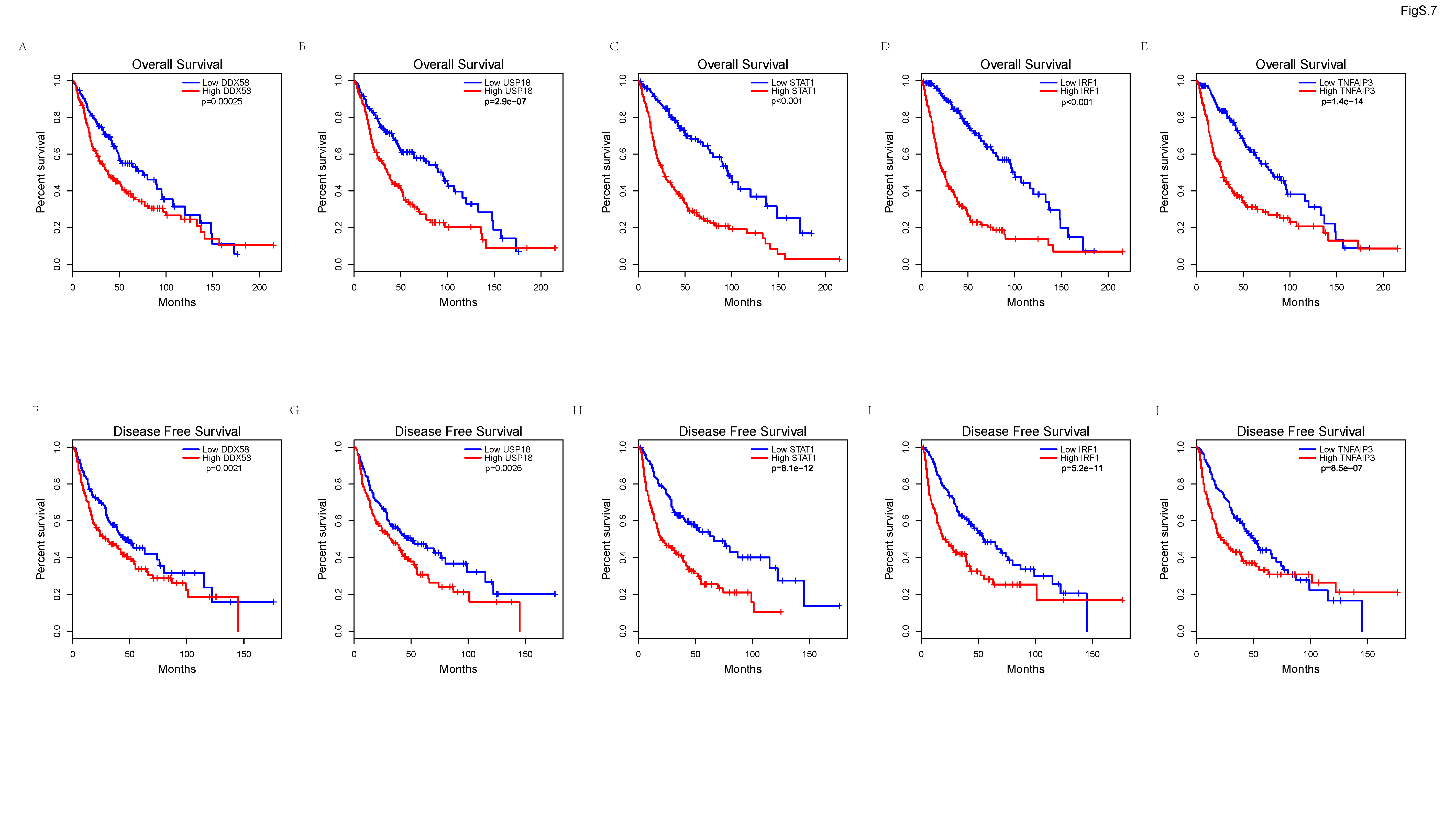

Supplement: Supplementary file 10 — Supp Figure 7 [file 41419_2023_5892_MOESM10_ESM.tif]
