# Supplementary material for: Super-enhancer-driven lncRNA LIMD1-AS1 activated by CDK7 promotes glioma progression
Source: Cell Death Dis. 2023 Jun 29;14(6):383. doi: 10.1038/s41419-023-05892-z (PMC10310775; doi:10.1038/s41419-023-05892-z)
Supplement: Supplementary file 2 — Supplement [file 41419_2023_5892_MOESM2_ESM.docx]

**Supplementary Table 1. Clinic-pathological characteristics of 43 glioma patients**

| Characteristic | n of patients |
| --- | --- |
|  |  |
| KPS |  |
| >80 | 34 |
| <80 | 9 |
| Gender |  |
| Male | 21 |
| Female | 22 |
| Age |  |
| ≤ 50 | 11 |
| > 50 | 32 |
| Grade |  |
| Low grade | 18 |
| GBM | 25 |

**Supplementary Table 2. Primers used for the construction of interference**

| Primer name | Sequence | |
| --- | --- | --- |
| Si-CDK7 | CAACAUUGGAUCCUACAUA | UAUGUAGGAUCCAAUGUUG |
| Si-LIMD1-AS1-1 | GCACCUGAGAAGUCUGUAATT | UUACAGACUUCUCAGGUGCTT |
| Si-LIMD1-AS1-2 | GGAUCUAACCAGCAGCGAATT | UUCGCUGCUGGUUAGAUCCTT |
| Si-HSPA5 | AGUGUUGGAAGAUUCUGAU | AUCAGAAUCUUCCAACACU |

**Supplementary Table 3. Primer pairs for promoter and enhancer regions in ChIP-qPCR**

|  | Forward Primer | Reverse Primer |
| --- | --- | --- |
| LIMD1-AS1-Pro | AACGGACCTACAGCAGCAAT | CAGGCTTCGGTACCCAGATA |
| LIMD1-AS1-SE1 | CTCCCAGCACATCCAAGAAT | TTTGGGCGTTATCAATCAGA |
| LIMD1-AS1-SE2 | GGCTTTCGTGGCAGAGAATA | CCCAACAGCCAAGAGGATTA |
| LIMD1-AS1-SE3 | GATTCCTGTTGGCCAGAAAC | ATGACTCCTGAGGCACTGCT |
| LIMD1-AS1-SE4 | TTTTTGCCTGGTTGTCATCA | CAACAGGATCTTACGCAGCA |
| LIMD1-AS1-SE5 | AGCTGTCGAATTGTCCGAAT | CCTTTAGCATCTGCCCACTC |

**Supplementary Table 4. Primers used for RT-qPCR**

| **RT-qPCR** | | |
| --- | --- | --- |
| Primer name | Forward Primer | Reverse Primer |
| LIMD1-AS1 | CAAAGCGCCCTGTTCCCAAT | ATCATCCGGTTGCTAACCTCC |
| CDK7 | ATGGCTCTGGACGTGAAGTC | CTTAATGGCGACAATTTGGTTG |
| HSPA5 | CGGTCTACTATGAAGCCCGT | CGAGCCACCAACAAGAACAA |
| STAT1 | AGTTCGGCAGCAGCTTAAAA | CACCACAAACGAGCTCTGAA |
| USP18 | CGAGAAAGATCTGCCACTCC | AGCTCATACTGCCCTCCAGA |
| TNFAIP3 | ATGCACCGATACACACTGGA | CACAAGCTTCCGGACTTCTC |
| IRF1 | AAGTCCAGCCGAGATGCTAA | AGTGGAGCTGCTGAGTCCAT |
| DDX58 | TGTCCACCTTCAGAAGTGTCT | AGCAGGCAAAGCAAGCTCTA |
| MALAT1 | GGATCCTAGACCAGCATGCC | AAAGGTTACCATAAGTAAGT |
| GAPDH | GTCGGAGTCAACGGATT | AAGCTTCCCGTTCTCAG |

**Supplementary Table 5 . Antibodies**

| GRP78 | 11587-I-AP(Proteintech) |
| --- | --- |
| CDK7(R) | 27027-I-AP(Proteintech) |
| CDK7(M) | 2916T(Cell Signaling Technology) |
| β-Actin | 66009-I-Ig(Proteintech) |
| MED1 | ab181103(Abcam) |
| MED1 | A1724(ABclonal) |
| Cyclin B1 | 12231 (Cell Signaling Technology) |
| Bcl-2 | 3498 (Cell Signaling Technology) |
| N-cadherin | 13116 (Cell Signaling Technology) |
| ZEB1 | 83243 (Cell Signaling Technology) |

**Supplementary Table 6. The sequences of the sgRNA**

| sgRNA | Sequence |
| --- | --- |
| LIMD1-AS1-SE4 | CTGCACTCACAACTCGAGGG |
| LIMD1-AS2-SE5 | ATTTAGTGCGCCGCCATGGG |

**Figure legends**

**Figure S1** (A) Expression levels of LIMD1-AS1 in normal brain and glioma samples from GTEX and TCGA. Each point represents one tissue sample. **p<0.01. (B)Expression levels of PVT1 in normal and tumor samples from GTEX and TCGA. **p<0.01.

**Figure S2** (A) Methylation levels of LIMD1-AS1 in normal brain and tumor samples from TCGA. (B)The correlation of LIMD1-AS1 expression and the methylation of LIMD1-AS1 in the TCGA database. (C) The copy number of LIMD1-AS1 glioma samples from TCGA. ***p<0.001 (D)The correlation of LIMD1-AS1 expression and the copy number of LIMD1-AS1 in the TCGA database.

**Figure S3** (A) The correlation between LIMD1-AS1 and SE complexes in glioma tissues from the CGGA database. (B) Heatmap of differentially expressed SE complexes between GBM and LGG in CGGA database. (C) Univariate analysis presents the hazard ratios and P-value of SE complexes-related genes by the forest plot. (D-E) Expression of CDK7 and MED1 in LN-18 and T98G cells transfected si-CDK7 as measured by RT-qPCR. **P < 0.01. (F) The correlation between LIMD1-AS1 and MED1 in glioma tissues.

**Figure S4** (A) Expressed HSPA5 between GBM and LGG in CGGA database. ***p<0.001. (B) Relative expressed HSPA5 in different grade of glioma in CGGA database. ***p<0.001. (C)The expression of LIMD1-AS1 is significantly associated with HSPA5 expression.

**Figure S5** (A-B) Relative expression of LIMD1-AS1 is significantly associated with interferon signaling expression in TCGA and CGGA database.

**Figure S6** (A-E) The expression of LIMD1-AS1 co-expression with IFN signatures (DDX58, USP18, STAT1, IRF1, and TNFAIP3) in TCGA Database. (F-J)The expression of LIMD1-AS1 co-expression with IFN signatures (DDX58, USP18, STAT1, IRF1, and TNFAIP3) in CGGA Database.

**Figure S7** (A-E) Overall survival of patients with glioma with IFN signatures expression levels estimated using the Kaplan-Meier method and compared with the log-rank test. (F-J) Disease-free survival of patients with glioma with IFN signatures expression levels estimated using the Kaplan-Meier method and compared with the log-rank test.
